# Widespread repression of anti-CRISPR production by anti-CRISPR-associated proteins

**DOI:** 10.1093/nar/gkac674

**Published:** 2022-08-10

**Authors:** Saadlee Shehreen, Nils Birkholz, Peter C Fineran, Chris M Brown

**Affiliations:** Department of Biochemistry, University of Otago, PO Box 56, Dunedin 9054, New Zealand; Department of Genetic Engineering & Biotechnology, University of Dhaka, Dhaka 1000, Bangladesh; Department of Microbiology and Immunology, University of Otago, Dunedin 9016, New Zealand; Bioprotection Aotearoa, University of Otago, PO Box 56, Dunedin 9054, New Zealand; Department of Microbiology and Immunology, University of Otago, Dunedin 9016, New Zealand; Bioprotection Aotearoa, University of Otago, PO Box 56, Dunedin 9054, New Zealand; Genetics Otago, University of Otago, PO Box 56, Dunedin 9054, New Zealand; Department of Biochemistry, University of Otago, PO Box 56, Dunedin 9054, New Zealand; Genetics Otago, University of Otago, PO Box 56, Dunedin 9054, New Zealand

## Abstract

Many bacteria use CRISPR-Cas systems to defend against invasive mobile genetic elements (MGEs). In response, MGEs have developed strategies to resist CRISPR-Cas, including the use of anti-CRISPR (Acr) proteins. Known *acr* genes may be followed in an operon by a putative regulatory Acr-associated gene (*aca*), suggesting the importance of regulation. Although ten families of helix-turn-helix (HTH) motif containing Aca proteins have been identified (Aca1-10), only three have been tested and shown to be transcriptional repressors of *acr-aca* expression. The AcrIIA1 protein (a Cas9 inhibitor) also contains a functionally similar HTH containing repressor domain. Here, we identified and analysed Aca and AcrIIA1 homologs across all bacterial genomes. Using HMM models we found *aca*-like genes are widely distributed in bacteria, both with and without known *acr* genes. The putative promoter regions of *acr-aca* operons were analysed and members of each family of bacterial Aca tested for regulatory function. For each Aca family, we predicted a conserved inverted repeat binding site within a core promoter. Promoters containing these sites directed reporter expression in *E. coli* and were repressed by the cognate Aca protein. These data demonstrate that *acr* repression by Aca proteins is widely conserved in nature.

## INTRODUCTION

Bacteriophages and other mobile genetic elements (MGEs) such as plasmids and genomic islands have a dynamic relationship with their bacterial hosts (1). Both MGEs and bacteria need quick adaptation strategies to increase their fitness in hostile environments (2,3). Lytic phages are an immediate threat to their hosts, as infection results in bacterial cell lysis. On the contrary, prophages typically integrate into the host chromosome and can have either positive or negative effects on host fitness (4,5). For example, prophages can increase fitness by introducing pathogenicity and virulence genes and conferring protection against other phages via superinfection immunity (6).

CRISPR-Cas systems provide adaptive immunity to prokaryotes (7). These systems have two major components: Clustered Regularly Interspaced Short Palindromic Repeats (CRISPRs) and their CRISPR-associated (Cas) proteins. Upon acquisition of short sequences from intruders into a heritable CRISPR array, which serves as a ‘memory bank’ containing spacers from a range of prior exposures, CRISPR-Cas systems offer resistance against those foreign elements (8–10). After transcription and processing of the array, the resulting short CRISPR RNAs (crRNAs) guide Cas proteins to recognize and cut foreign nucleic acids (11).

**Figure 1.**
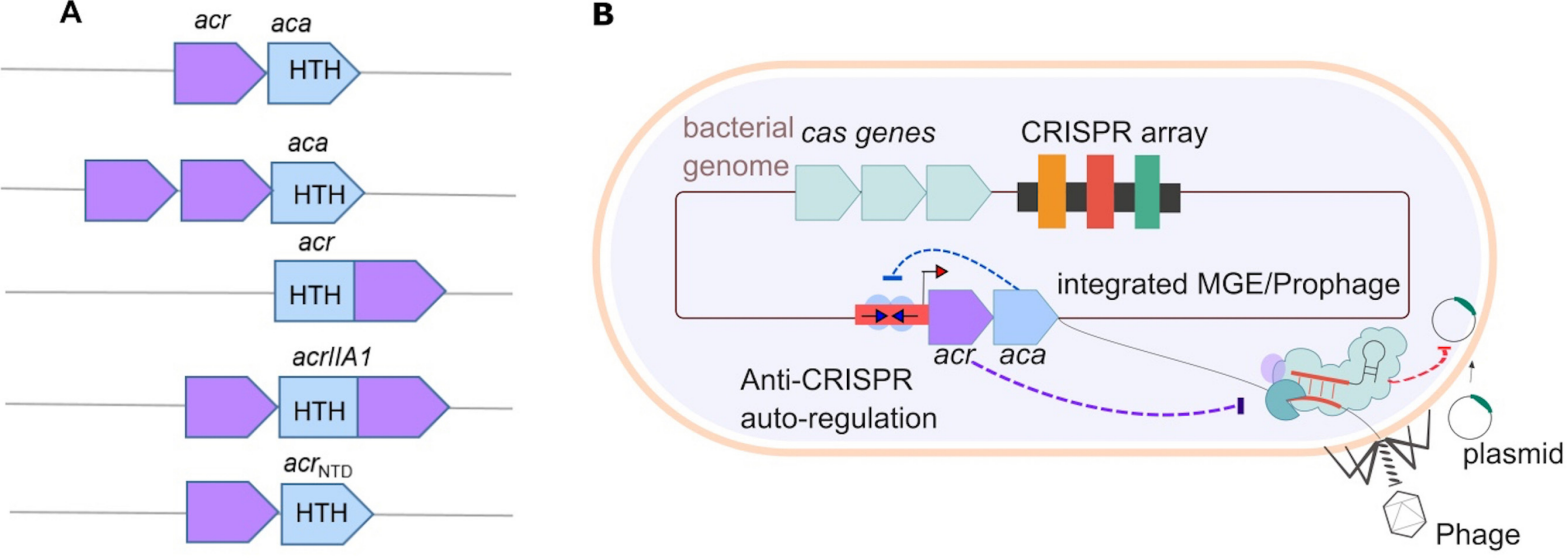
(**A**) Different organizations of *acr* operons. *acr* genes (purple) may be organized into operons with an *aca* (blue). The *aca* genes encode proteins that contain a helix-turn-helix (HTH) DNA binding motif. Single or multiple *acr* genes may cluster in one operon with an *aca* gene. Some *acr* genes encode the HTH motif within the *acr* (e.g*. acrIIA1*). (**B**) Model of autoregulation of Acrs. Bacteriophages and integrated mobile genetic elements (MGEs) have *acr-aca* operons. These invaders inhibit host immunity (CRISPR-Cas) by producing Acr proteins (purple). The Aca proteins (blue) bind to inverted repeats (arrows) which lie within the promoter region (red) and auto-regulate *acr* expression.

To overcome CRISPR-Cas immunity, some phages produce anti-CRISPR (Acr) proteins. These proteins inhibit CRISPR-Cas activity and allow phages to successfully propagate in their hosts (12–15). Acr-encoding loci vary in location and composition. Mobile genetic elements can include loci with one or more *acr(s)* in an operon (Figure 1). Interestingly, these loci often have genes encoding predicted DNA-binding helix-turn-helix (HTH) proteins downstream of the *acrs*, known as Acr-associated (Aca) proteins (14–17). The co-occurrence of *aca* genes with *acr* suggested their importance in regulating *acr* gene expression. Indeed, it was recently demonstrated that Aca1 and Aca2 recognize specific sequences overlapping with their cognate core *acr-aca* promoters and bind to those regions via their HTH domains. This interaction leads to transcriptional repression, presumably through blocking of RNA polymerase recruitment (18,19). Recent biochemical and structural studies showed the Aca1 and Aca2 proteins bind to palindromic DNA sequences in their promoter (20,21). Aca3 also acts to repress the promoter of its operon (18).

Some Acr proteins have a HTH domain within the same protein (16,22–25). These bi-functional Acr-Aca proteins can also regulate expression (22–24). For example, AcrIIA1 contains an N-terminal HTH domain, and this protein regulates an operon containing itself and several *acr* genes (15,22). For *acrIIA1* in *Listeria* phages and the *acrIF1-aca1* operon of *Pseudomonas* phage JBD30, *acr* repression is critical for phage replication, providing a reason for this regulation (18,22).

As well as for phages, the *acr-aca* operons of integrated MGEs may also confer benefits to the host (16,26,27). However, *acr* locus regulation in chromosomal and extrachromosomal MGEs has yet to be explored. Here, we performed a comprehensive analysis of Aca and AcrIIA1 homologs across bacteria to investigate if regulatory strategies or mechanistic similarities are shared amongst these Acr regulators (22,23). Examination of Aca sequences revealed interesting similarities and differences between Acas. Table 1 provides a summary of information about these Aca proteins and AcrIIA1 (14–17,19,20,22–24,26–30).

**Table 1.** Summary of Aca protein characteristics

Aca	Organism^a^	Accession^a^	Size^a^ (aa)	Pfam domain^a,b^	Associated Acr^a,b^	Closest structures (Confidence, %id), [Coverage % (residues)]^b^	References
**Aca1**	*Pseudomonas* phage JBD30	YP_007392343	79	HTH_24	I-E and I-F	Aca1 (7FA3)	(13–15,28)
				HTH_8		(100, 99%), [73% (21–79)]	
				HTH_XRE			
				HTH_31			
**Aca2**	*Oceanimonas smirnovii*	WP_019933869.1	125	DUF1870	I-F	Aca2 (7B5J) (100,100%), [99%,1–124]	(13–15,28)
**Aca3**	*Neisseria meningitides*	WP_049360086.1	70	HTH_XRE	II-C	HTH-type transcriptional regulator MqsA (3FMY)	(13–15,28)
				HTH_3		(98.1, 27%) [78%(12–67)]	
				HTH_19			
**Aca4**	*Pseudomonas aeruginosa*	WP_071533911.1	67	HTH_23	I-F	TrfB transcriptional repressor protein (2W7N)	(27,28)
				KORA		(97.4, 29%) [88%(1–60)]	
**Aca5**	*Pectobacterium carotovorum*	WP_039494319.1	60	HTH_28	I-F	Repressor Rep-Ant complex from *Salmonella*-temperate phage (5D50)	(27,28)
				HTH_3		(97, 26%),[55%(3–36)]	
**Aca6**	*Alcanivorax* sp.	WP_035450933.1	65	HTH_3	I-F	Antitoxin Iba-2 (5J9I) (96.8, 24%), [83%(4–58)]	(27,28)
**Aca7**	*Halomonas caseinilytica*	WP_064702654.1	68	HTH_3		Regulatory protein C (4YBA) (94.2, 20%), [77% (11–64)]	(27,28)
**Aca9**	*Klebsiella pneumoniae* plasmid	QBI37412	69	HTH_3^2^	I-F	HigA2 antitoxin C-terminal domain (5J9I) (95.4, 23%), [68%(5–52)]	(12)
**Aca10**	*Pseudomonas citronellolis*	WP_074980464.1	65	HTH_31^4^	I-C	HigBA2 toxin-antitoxin complex (5JAA) (99.5, 30%), [84%(6–61)]	(26)
**AcrIIA1**	*Listeria monocytogenes*	WP_003722518.1	149	HTH_26	II-A	Toxin-antitoxin complex GraTA (6F8S)	(22,23,31)
				HTH_3		(94.2, 21%), [46% (4–76)]	

^a^Information taken from previous studies, references in the text.

^b^Found in this study. The most closely related PDB structures were predicted using Phyre2.

All Aca have predicted HTH DNA-binding domains. However, the HTH domains belong to different Pfam families. For example, Aca3, Aca5, Aca6, Aca7, Aca9, AcrIIA1 have HTH_3 domains whereas Aca2 has a DUF1870 domain. Some of the Aca homologs are associated with known, candidate, or putative *acr* genes and are likely regulators, while others occur solitarily and may represent anti-anti-CRISPRs, in that they could repress the expression of incoming MGE-encoded anti-CRISPRs (22,23).

In addition, we computationally analysed the putative regulatory regions of *acr-aca* operons from bacterial genomes and predict motifs that could be binding sites of Aca proteins. To test the regulatory effects of Aca, we selected a representative Aca-promoter pair from each of the ten families and demonstrated that in all cases these proteins act as transcriptional repressors. Overall, this study demonstrates the widespread nature of Acr regulation by Aca proteins and that these function to repress *acr-aca* operon expression.

## MATERIALS AND METHODS

### Hidden Markov models of Aca1–10 and AcrIIA1

A Position-Specific Iterative Basic Local Alignment Search Tool (PSI-BLAST) (32) search was performed with default parameters against the non-redundant protein database (NCBI-NR; retrieved October 2018) using the Aca protein accession numbers listed in Table 1 as the query. Aca (Aca1–Aca10) and AcrIIA1 homologs were identified through PSI-BLAST (four iterations). As there was no single query coverage and sequence identity threshold for these Aca families [11 in total; (Aca1- Aca10) and AcrIIA1], the parameters and values for each of the PSI-BLAST searches were set after manually checking the individual alignment between the query and the corresponding hits. Additionally, the homologs were selected based on the annotation of the proteins [Electronic Supplement S1]. Potential bias of the HMMs from highly similar sequences were excluded by choosing a small number of dissimilar homologs for some Aca families. The query covers, identity, and e-value for each of the searches are summarized in Supplementary Table S1. Respective PSI-BLAST (32) hits were used to build Hidden Markov models (HMM) of individual Aca (and AcrIIA1) proteins by using HMMER (v3.0.0) (33).

### Identification of Aca & AcrIIA1 homologs in the NCBI-NR database

An HMM search was performed (e-value cut-off: 10^–9^) against the non-redundant protein database (NCBI-NR; retrieved September 2020) to identify all homologs [Electronic Supplement S2]. The non-redundant protein sequences were generated by using CD-HIT (https://www.weizhongli-lab.org/cd-hit/) (34). The similarity threshold was set to 0.8. Within this similarity threshold, we found non-redundant homologs from diverse bacterial species. The Aca proteins of Table 1 were then used as a query to perform a similarity search (blastp with default parameters) against the NCBI non-redundant protein database (Electronic Supplement S2).

### Gene association networks

**Figure 2.**
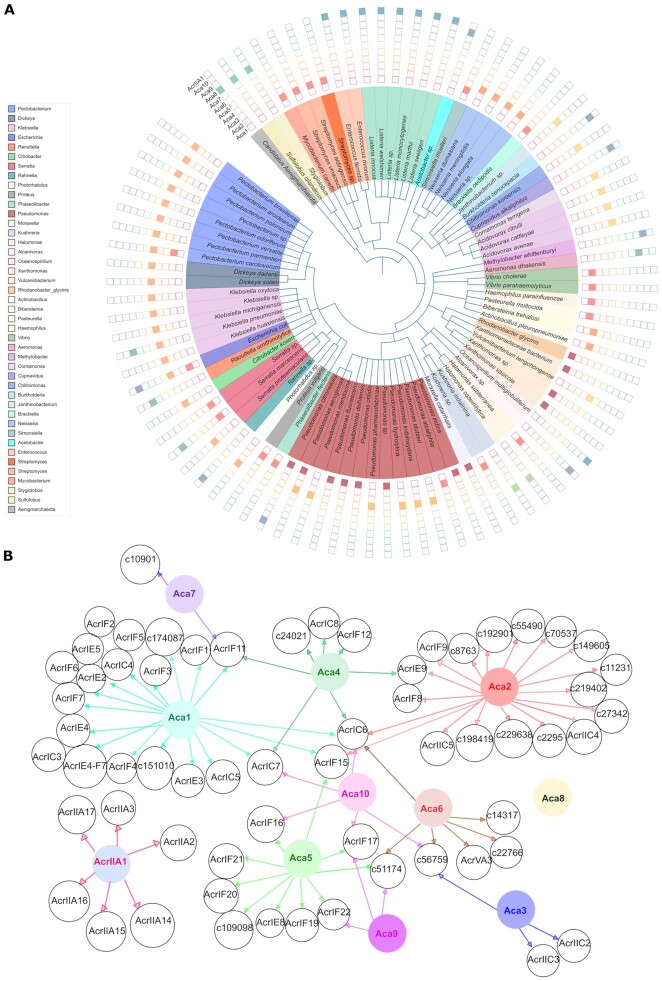
Overview of Aca homologs. (**A**) The distribution of known and candidate Acr-associated Aca proteins (*n* = 353) in different bacterial species and archaeal viruses. Filled squares represent the presence of the indicated Aca family in different bacterial genomes, phages, prophages and archaeal viruses. (**B**) Gene association network showing the genetic link between known or candidate Acr with Aca1–Aca10 and AcrIIA1 homologs. Different colours are assigned for different Aca (and AcrIIA1). The arrows indicate associations by genetic context.

Reference genomes of RefSeq bacteria were obtained from RefSeq201 (July 2020) [downloaded from https://ftp.ncbi.nlm.nih.gov/refseq/release/]. The sequences from each of these bacterial strains contain contigs or scaffolds of the chromosomes and may also contain contigs or scaffolds from episomes. The Aca (and AcrIIA1) homologs were used to search the translated genomes by tblastn (*e*-value cutoff: 10^–6^). Then, hits from each of the Aca (and AcrIIA1) groups were filtered based on bit score (range: 100–250). The bit score thresholds were set after systematically checking the individual alignments between the query and the corresponding hits. Within the threshold, the hits were distributed over different species of bacteria. A perl script was used to extract 5000 bp upstream and downstream from each tblastn hit. We aimed to analyse the *acr-aca* operons and promoters from non-redundant sequence sets, eliminating very similar sequences. These non-redundant sequences were generated by using CD-HIT-EST (https://www.weizhongli-lab.org/cd-hit/) (34) and the similarity threshold was set to 0.9. The ∼10 kb of extracted regions were then annotated with Prokka implemented in Galaxy (v1.11) (http://galaxy.otago.ac.nz:8080/) (35). Prokka was used with all Aca (and AcrIIA1) homolog sequences, known and candidate Acrs, and HMMs of Aca (and AcrIIA1) families. Genbank format files (.gb) [Additional data github] generated by Prokka were analysed with Geneious prime (v2019.1.3) to view co-occurrence of Acr and Aca homologs. To obtain a gene association network between *acr* and *aca* genes, an R script was used in RStudio Server (v1.3.1093) http://www.rstudio.com/ [Electronic Supplement S5]. Figure 2B has been adjusted manually to ensure readability.

### Analyses of Aca & AcrIIA1 homologs

The HTH domains were predicted by Pfam searches (36). The secondary structure of the Aca and AcrIIA1 proteins was predicted and analysed with Phyre2 (37).

### Phylogenetic analyses of Aca and AcrIIA1 proteins

Yin *et al.* (28) built a phylogenetic tree with 39 homologs of Aca1-Aca8 and AcrIIA1. These proteins were used to construct a phylogenetic tree. Additionally, the homologs of Aca9 and Aca10 were identified through PSI-BLAST (four iterations; cut-off: query covers 80–100%, identity 50–100%, e-value 0.0001) with default parameters against the non-redundant protein database (NCBI-NR; retrieved May 2020). The Aca9 and Aca10 protein accessions used are listed in Table 1. Only the N-terminal regulatory domain of AcrIIA1 was used, rather than full-length protein including the Acr domain. Protein sequences were aligned with the MUSCLE algorithm [Electronic Supplement S6] (38) by using the Neighbor-joining clustering method (maximum number of iterations: 500). The aligned sequences were then used to build an approximately-maximum-likelihood phylogeny using Fast Tree [Electronic Supplement S7] (39). The tree was displayed with Geneious Prime (v2019.1.3) and iTOL(v5) (40). All the software used in this study are listed in Supplementary Table S7. Based on the NCBI taxonomy, PhyloT (v2) (https://phylot.biobyte.de/) was used to generate a phylogenetic tree of 91 representative bacterial species and archaeal viruses (Figure 2A). The presence and absence of 353 Aca homologs in bacterial species (*n* = 339) and archaeal viruses (*n* = 14) classified into 11 types were annotated on the tree using iTOL (v5) https://itol.embl.de/ (40).

### Identification of *aca* (and *acrIIA1*) promoters

The putative promoter regions of *acr-aca* or *acrIIA1* operons and standalone *aca* were extracted. Potential operons were identified and 400 bp upstream was extracted using the CDS feature coordinates in GenBank format files from prokka output or from NCBI. CDS on the same strand with 55 bases or less between CDS were considered to be operons (41).

### Analysis of promoter regions

Common motifs (Electronic Supplement S3 and Figure 4) from the high confidence promoter sequence sets were collected by MEME Suite (v5.2.0) (42). The motifs were searched for in all promoter sequences [Supplementary Table S3] using MAST (42). Potential –35 and –10 sites in the promoter regions were predicted by BPROM (SoftBerry) (43). The DNA sequence analysis of the promoter regions was performed with Geneious Prime (v2019.1.3) and MUSCLE (38) was used for sequence alignment. All motifs were compared using Tomtom [Electronic Supplement S4] (44) (*P*-value cut-off < e-01 and distance measure Pearson correlation coefficient).

### Cloning of candidate promoters and *aca* genes

Candidate promoters of *acr-aca* operons (Electronic Supplement S8 and Figure 4) and corresponding *aca* genes were synthesised as gBlocks (IDT) [Supplement Table S4]. The candidate promoters were cloned into the SpeI and PstI sites of the pGR2 vector so that the promoter directed the synthesis of Red Fluorescent Protein (RFP). The pGR2 vector contains the GFP and RFP encoding cassette from pGR cloned into the pHERD30T shuttle vector (45). Aca-encoding genes were ligated into the NcoI and HindIII sites of pCDF-1b and transformed into competent *E. coli* DH5α [Supplementary Table S5]. The sequence was confirmed by Sanger sequencing using primers outside of the cloning sites [Supplementary Table S4]. A list of the oligonucleotides, bacterial strains and plasmids used in this study can be found in Supplementary Tables S4–S6. Restriction digests, ligations, *E. coli* transformations and agarose gel electrophoresis were performed using standard techniques.

### Preparation of *E. coli* BL21 strains for *aca* functional testing

The pGR2 plasmids with different candidate promoters and pCDF-1b plasmids with corresponding *aca* genes or empty pCDF-1b were electroporated into *E. coli* BL21using a Bio-Rad GenePulser Xcell system (set to 2500 V, 25 μF, 200 Ω) in Bio-Rad electroporation cuvettes with a 0.1 cm electrode gap, followed by 2 h recovery in LB medium at 37°C at 180 rpm. The positive clones were selected by spreading onto LB agar plates containing 30 μg/ml gentamicin and 50 μg/ml streptomycin.

### *In vitro* reporter assay

Promoter activity was measured with *rfp* under control of the acr–aca promoter(s). The Aca protein was induced by IPTG. To determine the effect of Aca expression on *acr–aca* promoters, each *rfp* reporter plasmid was tested with an Aca expression plasmid (+Aca; Supplementary Table S6) with and without IPTG induction, or the corresponding empty vector (–Aca; pCDF-1b). A single colony of *E. coli* BL21 containing the desired plasmids was used to inoculate 500 μl of LB medium containing the appropriate antibiotics, and IPTG was added to a final concentration of 50 μM. After 24 h of growth in a 96-well plate in an IncuMix incubator shaker at 700 rpm at 37°C, fluorescence of plasmid-encoded RFP was measured using a plate reader (CLARIOstar plus; BMG LABTECH). Blank-corrected fluorescence intensity of RFP (excitation wavelength: 562–12 nm, emission wavelength: 603–20 nm, Gain setting:1000) was measured for at least six biological replicates.

## RESULTS

### Aca like genes are widespread and not always associated with known *acrs*

Previous studies suggested that Aca families had some common, yet distinct, features—in particular a range of HTH domains (Table 1). To further understand the role of Aca proteins, representative members were analysed for structural similarity using Phyre2. Interestingly, three members of the Aca family (Aca6, Aca9 and Aca10) and AcrIIA1 proteins have similarities to the HTH-containing antitoxins/repressors HigA2 and GraA, respectively (Table 1). More detail of each Aca protein family is provided in the Supplementary Text file.

To identify Aca homologs across bacterial taxa, HMMs of each Aca (and AcrIIA1) family were built and used to search bacterial genome sequences (Materials and Methods). As HTH domain-containing proteins are ubiquitous in nature (over 2 million proteins have Pfam HTH domains), stringent criteria were used to reduce false-positive hits (Materials and Methods). These models defined each of the Aca families and closely related proteins. The association of the predicted *aca* genes with *acr*s in bacterial MGEs was determined by extracting the upstream and downstream sequences (∼5 kb) and predicting *acr*s in these regions (Materials and Methods). Unexpectedly, among 2599 non-redundant Aca-like homologs, only 386 (or 15%) from diverse bacteria were associated with known (*n* = 276) Acrs. The number increased slightly if previously predicted candidate (*n* = 63) or ‘putative’ (*n* = 47) Acr-like proteins were included (29). In the case of Aca2 (DUF1870), only 3% (35 of 1223 significant hits) of Aca2-like proteins are found with upstream *acr* genes in bacterial genomes [Supplementary Figure S1]. Previously, structural homologs of Aca2, such as YdiL from *Salmonella*, had been identified without *acr* in pathogenic bacteria (20).

For the *aca*s that were clearly associated with *acr*s, a wide phylogenetic distribution of homologs was found across bacterial genera including *Pseudomonas, Neisseria, Pectobacterium, Klebsiella, Serratia*, *Listeria* and *Escherichia* (Figure 2A). Interestingly, in this large dataset, we observed Aca1, Aca3, Aca5, Aca6, Aca7 and AcrIIA1 homologs were grouped in clades of related species (Figure 2A). Aca4 is found only in different *Pseudomonas* species (Figure 2A). On the other hand, Aca2, Aca9 and Aca10 homologs were distributed in distantly related species. Although Aca2 and Aca9 proteins were distributed across diverse species (Figure 2A), we found these genes were phylogenetically related [Supplementary Figure S2], suggesting potential dissemination across species via horizontal gene transfer. Within a bacterial genus there can be more than one Aca protein. For example, Aca1, Aca2, Aca4 and Aca10 families are found in *Pseudomonas*, the most studied genus for Aca or Acr discovery (Figure 2A).

Yin *et al.* analysed 39 homologs of the families known then (Aca1-Aca8 and AcrIIA1) and observed grouping of Aca proteins into nine monophyletic families (28). We next sought to assess the relatedness of the Aca protein families by examining the similarities among our expanded set of Aca proteins (*n* = 97). In agreement and extending the earlier work, Aca families (Aca1–Aca10 and AcrIIA1) formed distinct groups (Supplementary Figure S2). This also supports the idea that, despite having common HTH domains, the Aca families are indeed different (Table 1, Supplementary Figure S2). Overall, these data indicate diverse bacteria have evolved, or recruited, distantly related Aca proteins and then some (e.g. Aca2) have been transferred horizontally.

We were interested in whether specific Acas are genetically linked to specific Acr families (Figure 2B). The archaeal Aca8 was not found in bacteria and plasmids, so no association with other bacterial Acrs is shown. Many different Acrs were clustered with either Aca1 or Aca2. In addition to known Acrs, such as AcrIF8 and AcrIF9, genes encoding Aca2 clustered with many of the candidate Acrs predicted recently (29). For Aca1 and Aca2, the analysis may be biased by their early discovery and hence the greater number of known associated genes encoding Acr proteins. In contrast, Aca7 was only found with AcrIF11 and one candidate Acr protein.

It was interesting to observe that specific *acr* homologs co-occurred with different *aca* genes in different species. For example, AcrIF17 homologs were associated with Aca5, Aca9 and Aca10 in different species of *Pectobacterium, Serratia* and *Rahnella*, respectively. These data might indicate that evolution has grouped various combinations of *acr* and *aca* genes into operons in different species and that specific types of Acrs and Acas are not functionally coupled.

### The *aca*-like genes found with and without *acrs* have distinct features

**Figure 3.**
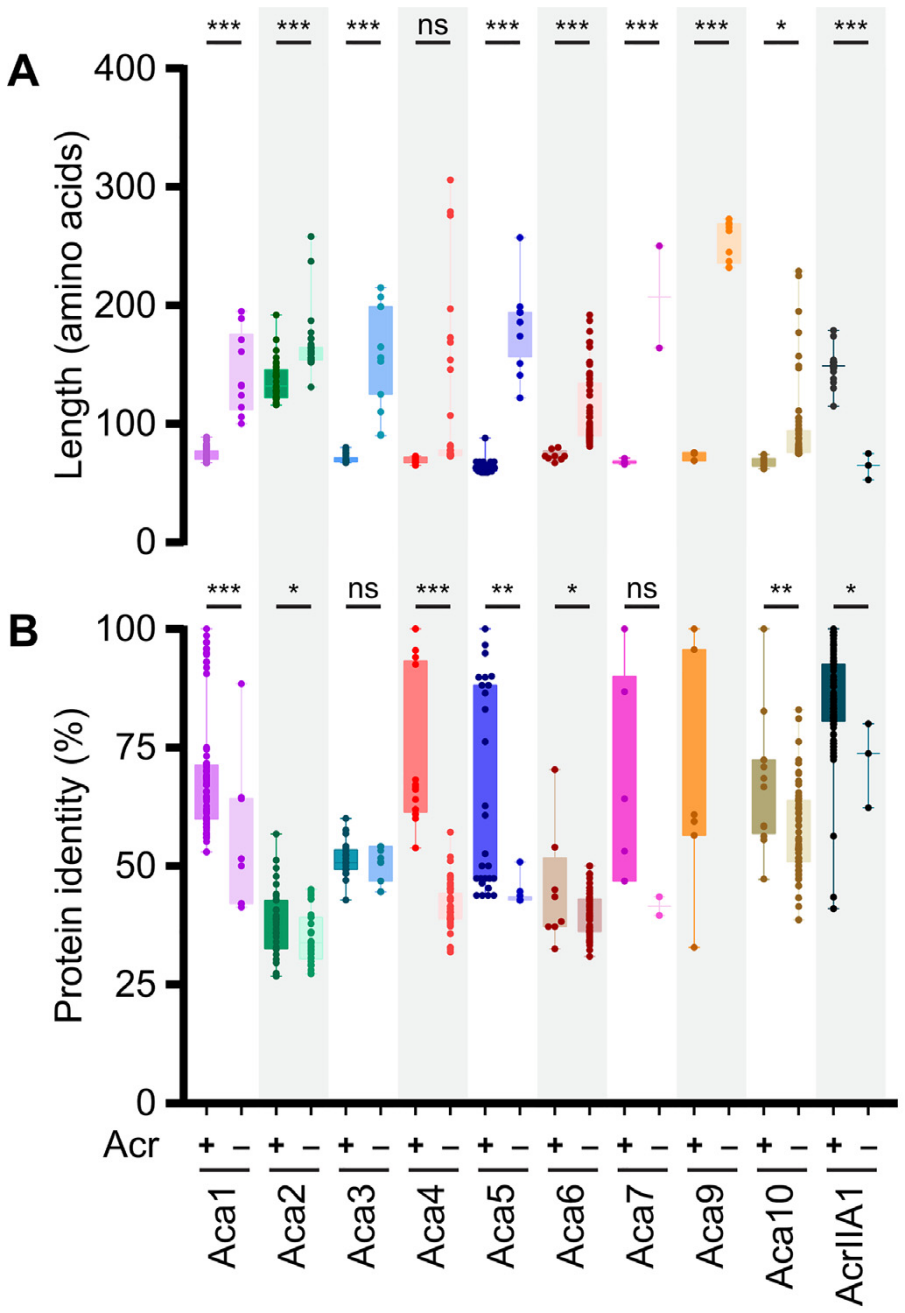
Aca proteins that are Acr-associated versus solo typically differ in length and amino acid identity (blastp). Comparison of length (**A**) and percentage amino acid identity (**B**) of Acr-associated (+) and solo Aca (–) proteins. The coloured box indicates means with the 95% confidence intervals. The numbers of homologs analysed in this study are listed in Supplementary Table S2. Only proteins exhibiting >40% query coverage to the reference proteins were used. Note that AcrIIA1 is a bi-functional Acr protein and the small homologs lack the Acr domain (22) and are counted as solo. Statistical significance was calculated by unpaired *t*-test (***P< 0.001, **P< 0.01, *P< 0.05, ns *P*> 0.05).

To compare the properties of the Aca families, we characterised them by protein length and within-family similarity. Aca proteins associated with Acrs mostly had mean sizes of 60–65 amino acids, indicating proteins with a single HTH domain (Figure 3A). However, Aca2 and AcrIIA1 homologs were longer (mean 136 and 149 amino acids, respectively). AcrIIA1 homologs are larger because they are Acr-Aca fusions (22). For Aca2, the longer C-terminal domain is responsible for the dimerization of the protein (20,21).

Most *aca*-like genes are found without nearby *acr* or other genes and the encoded proteins are here termed ‘solo Aca’. The majority of these were larger than the mean size of their corresponding Aca family (Figure 3A). For some Aca families (Aca2, Aca3 and Aca6) identities to the known Aca had similar ranges with or without Acr association (Figure 3B). However, for most Aca, those homologs that are not associated with *acr* genes were less similar to the known Aca. For example, the mean identity for the Acr-associated Aca4 was 72%, while the solo Aca4 homologs exhibit only 42% mean identity to the reference Aca4 protein listed in Table 1 (Figure 3B).

There was a group of large Aca9-like proteins (232–273 amino acids) that were not found downstream of known or candidate *acr* genes, but candidate *acr* genes were found within 5 kb around the *aca* locus. These proteins are widely distributed in a phylum called the CFB group. In addition to an HTH domain, these larger Aca9 proteins contained a predicted C-terminal S24 peptidase domain in several species [Electronic Supplement S2]. It is possible that these relatively large *aca* homologs may be Aca-Acr hybrids as shown for AcrIIA1.

### Putative Aca binding sites are typically located within the *acr-aca* promoter

**Figure 4.**
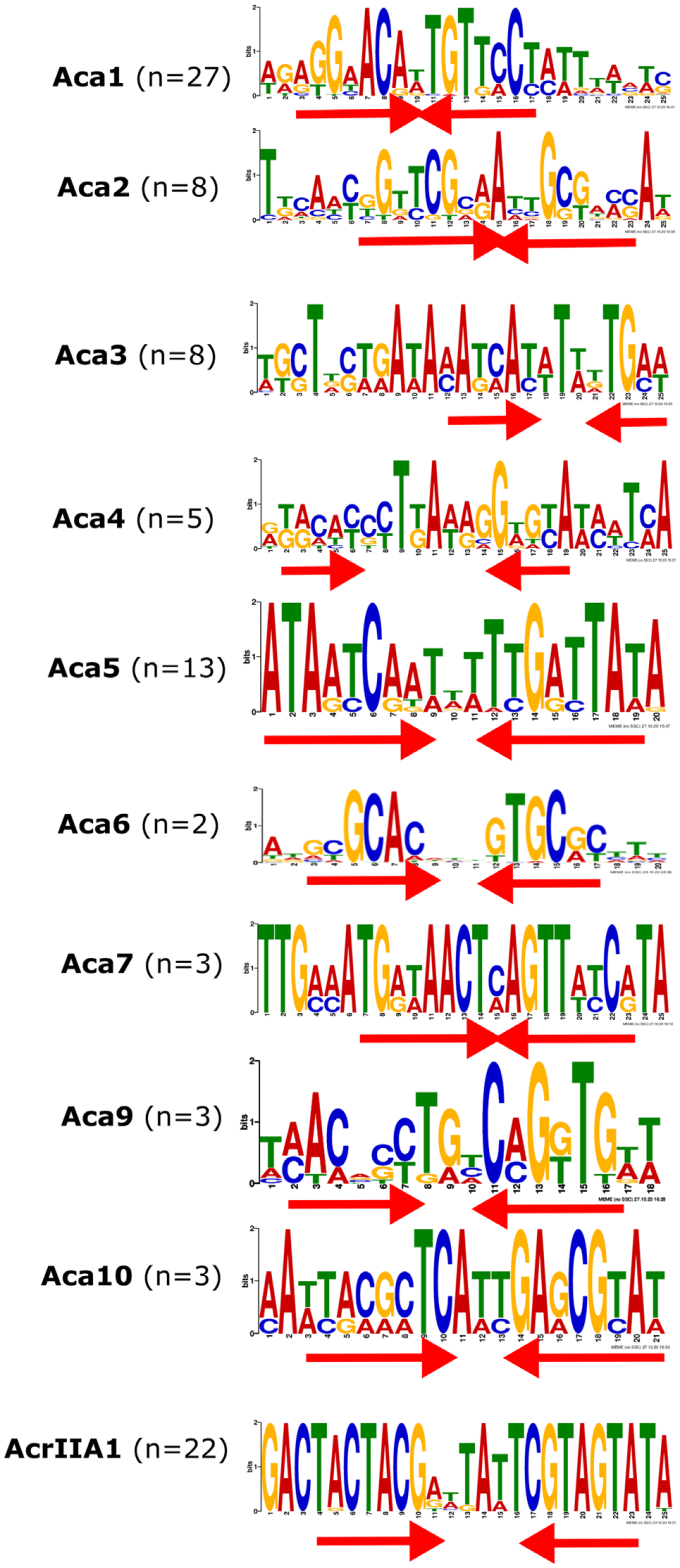
DNA binding sites within *acr-aca* promoters as predicted by MEME. Inverted repeats are indicated with red arrows. The number (*n*) indicates the number of the non-redundant promoter sequences with the site.

At the start of infection, many phages rely on the bacterial RNA polymerase to transcribe early genes. These early phage promoters resemble strong bacterial housekeeping promoters as they need to compete with host promoters for bacterial RNA polymerase (RNAP) (46). In agreement, promoter elements (with close similarity to the consensus –35 and –10 sequences) were previously found within the *aca1, aca2, aca3* and *acrIIA1*operons (18,19,22). To elucidate whether all Aca protein families and AcrIIA1 are likely to control the expression of the associated *acr* genes, we searched for potential regulator binding sites in the promoter. The putative promoters of all *acr-aca* operons were analysed. We initially analysed operons containing both *acr* and *aca* as a set of high-confidence promoters (Supplementary Table S3). For detection of DNA binding sites, we performed motif discovery using MEME and palindrome in 400 bp before the initiation codon [Material and Methods]. All significant predictions were within 150 bp and most within 120 bp. The most common (and conserved) motifs within 150 bp upstream of the *acr* initiation codon were analysed as putative Aca binding sites (Figure 4 and Supplementary Figures S3 and S4).

We found consensus –35 and –10 sequences in each of the ten high-confidence promoter sets (excluding the Aca8 family, since we found no examples in bacterial genomes). The core elements are distinct for each of the Aca proteins. Strikingly, we also observed one or two conserved inverted repeats (IR) sequences within each promoter region. One of the inverted repeats (here termed IR1) was always found within the predicted core promoter (Supplementary Figure S4), which is consistent with an auto-repression role for the Aca to exclude and block RNA polymerase recruitment (as previously shown for Aca1-3 (18,19). In several cases (Aca1, Aca2, Aca4, Aca5, Aca9 and Aca10), more than one IR was observed per promoter, with the additional IR here denoted IR2 (Supplementary Figure S4). In each case, IR2 was similar to the IR1 within the same promoter and was located either upstream or downstream of the consensus –35 and –10 sequences. However, most promoters have only one IR located within the core promoter. In summary, *acr-aca* operons have predicted constitutive promoters with one or two IRs that are predicted to enable Aca-mediated repression.

### Acas repress their cognate *acr-aca* promoters

**Figure 5.**
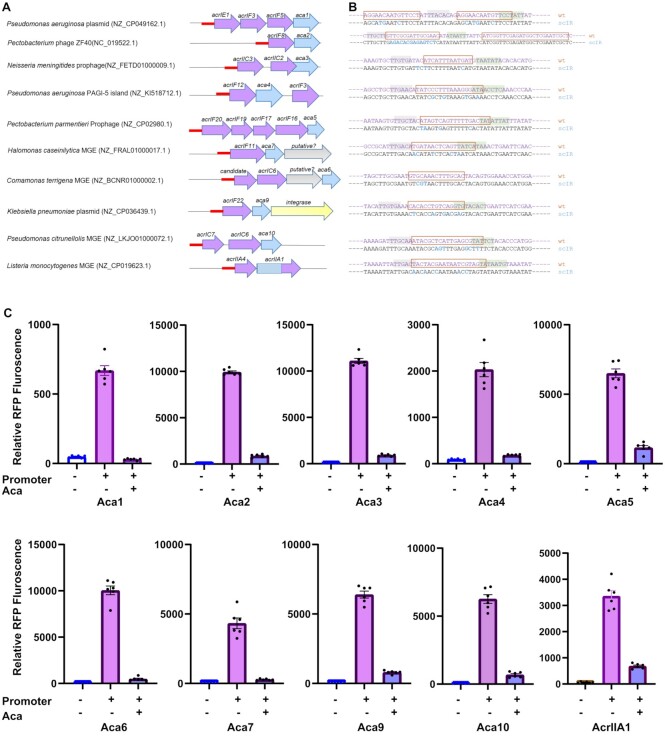
Repression by Aca proteins. (**A**) Schematic representation of tested promoters from diverse species. Ten promoter/Aca pairs were selected to test the effect of Aca proteins on the promoters. The promoters are indicated by red straight lines. (**B**) The wild type and mutated promoters are shown. Scrambled bases are shown in blue. (**C**) Aca proteins repress the cognate promoters. Activity of the promoter variants in *E. coli* BL21 in the presence and absence (+/–) of Aca proteins, determined as relative RFP fluorescence. In each graph, the RFP fluorescence values were normalized with the OD_600_ values. Data are presented as the mean ± SEM of six biological replicates, + or - denotes the presence or absence (empty vector) of the promoter or *aca* gene.

To determine whether the members of an Aca family repress expression from the *acr-aca* promoters, a plasmid-based fluorescent reporter assay in *E. coli* was used. For this study, we selected ten putative promoters of *acr-aca* operons from different mobile genetic elements found in different species (Figure 5A). All of these operons contain single or multiple *acr*s with different *aca* genes. The *acrIF8-aca2* promoter sequence from phage ZF40, and encoded Aca2 was used as positive control, as this combination had previously been examined in a different reporter assay (19). The remaining nine Aca/promoter pairs have not been tested previously, but some (Aca1, Aca3 and AcrIIA1) are orthologs of previously tested pairs (18,19,22). Putative *acr*-*aca* promoters,120 bases prior to the ATG as this region contained the predicted motifs (Figure 5A), were inserted upstream of a reporter gene (*rfp*) encoding RFP (red fluorescent protein). All tested *acr-aca* promoters enabled robust *rfp* expression in *E. coli* (Figure 5C, and Supplementary Figure S5), although they come from various Proteobacteria and Firmicutes (Figure 5B). This is consistent with our prediction that these are strong promoters in bacteria.

The corresponding *aca* genes were expressed from a separate plasmid (pCDF-1b), with expression induced by IPTG. When expression of the cognate Aca protein was induced (e.g. Aca3 and the *acr-aca3* promoter), reporter expression was significantly reduced by, on average, 90% compared with the control, which indicates strong repression of the *acr-aca* promoters by Aca proteins (Figure 5). To further examine the strength of the Aca-promoter repression, we also compared repression in the absence of IPTG induction of Aca repressor expression. As expected, without induction, we observed either no or weaker repression (mean 52%, Supplementary Figure S6), with the latter potentially due to low-level leaky *aca* expression.

To test whether the repression was due to each Aca binding to the predicted inverted repeat (IR) sites, we mutated the IR sequences (Figure 5B). In most cases, the mutation of bases did not disrupt the core promoter elements. However, in the mutated *aca10* promoter one of the conserved adenine bases of the predicted –10 site was changed with thymine. We observed poor expression of this mutated *aca10* promoter, likely due to this change. However, in most cases, scrambling of the predicted IR sites abolished repression by Aca proteins (Supplementary Figure S7), which provides evidence that repression occurred by binding of Aca proteins to the IR shown (Figures 4 and 5A). Despite the mutation, some repression by Aca9 was still observed. However, the mutations left the core motif (CACC) of the half site intact. Also, we observed the presence of the core motif upstream of –35 region. Taken together, we can conclude that the presence of the core motifs more than once in the *acr-aca9* promoters plays a significant role in regulation. In summary, diverse Aca proteins repress the expression of their operons through recognition of IR sequences in their promoter regions.

## DISCUSSION

This study investigated the regulation of *acr* gene expression, with a focus on the DNA-binding Aca proteins. The homology-based search conducted in this study identified many more Aca homologs, including *aca* genes not closely associated with known or predicted *acr*s. This is likely partly due to some *acr*s being unidentified and some *aca*-like genes having distinct functions.

The expression of *acr*s may need to be regulated in phages. Early in phage infection, viral *acr* operons can be expressed strongly from their promoters (30). The Acrs then perform their role to inhibit the CRISPR-Cas defence system (47–49). However, for some Acrs it has been shown that their strong expression needs to be downregulated to prevent detrimental effects on the host bacterium and phage (12,30). Also, when temperate phages are integrated as prophages and express *acr*s, they may confer a benefit in preventing autoimmunity by self-targeting CRISPR spacers (50). However, this must be balanced, as *acr* expression will also inhibit their host bacterium's CRISPR-Cas defence against invasion by competing phages.

We found that many *aca*s are predicted to be transcribed as single genes, without any other genes in an operon. This raises the possibility that the *aca*-like genes have different functions or may regulate foreign *acr* genes in *trans*. The solo *aca*-like genes are larger than their *acr*-associated counterparts. The encoded proteins may contain additional domains, such as the peptidase domain we identified in solo Aca9 homologs, possibly indicating additional bi-functional Acr-Aca proteins (12,22,28). However, the biological significance of these large solo Acas remains to be tested. The widespread presence of solo *aca* genes supports the idea that some bacteria may use these Aca proteins as anti-anti-CRISPRs, i.e. as repressors of foreign *acr* genes, as previously shown for the AcrIIA1 N-terminal and AcrVIA1 regulators (22–24,49). The use of anti-CRISPR repressors can help to overcome the impediments in CRISPR-based editing caused by prophage encoded Acrs (51).

We have shown that Aca(s) are distantly related to each other. Although they have a common helix-turn-helix DNA binding domains, these belong to different families. Interestingly, here we show that a few Aca proteins (Aca1, Aca2, Aca5) were associated with many Acr families whereas other Aca proteins (Aca3, Aca6, Aca7, Aca9, Aca10) were associated with few known Acrs. Intriguingly, we observed three out of ten Aca families and HTH containing AcrIIA1 exhibit structural similarities with two HTH containing regulatory antitoxin proteins HigA2 and GraA. These antitoxins are components of type II toxin-antitoxin (TA) systems, in which the toxin gene precedes that of the antitoxin (52–54), and act as autorepressors by binding operator sequences in their operons’ promoter. This operon organization is similar to most of the analysed *acr-aca* loci. Type II TA loci are often associated with MGEs (55), as are *acr-aca* loci (12,16). A previous study not only reported TA genes in the neighbourhood of the *acr-aca* but also found a significant number of putative *acr-aca* loci matched with TA in pairs (28). Moreover, Aca2 and the antitoxin MqsA possess structural similarity (19). Consequently, this suggests that TA and *acr-aca* could be linked by a common evolutionary origin, at least in some MGEs. In another study, Aca2 and YdiL (both from proteobacterial pathogens) were shown to be structurally similar but are likely involved in controlling different biological processes (20,21).

Conserved IRs were found within predicted core *acr-aca* promoter elements (–35 and –10). Some promoters (*aca1, aca2* and *aca4*) contained more than one IR sequence, as noted previously (18,19,22). In Aca1- and Aca2-regulated promoters these second IR sites are not essential for transcriptional repression (18,19). These binding motifs are not always present in the promoter regions of the solo Aca proteins, suggesting the presence of alternative binding sites or a different regulatory mechanism for these larger Aca homologs.

The Acr proteins themselves, or the process of their expression might be toxic to the bacterium or phage. Previous studies in *P. aeruginosa* showed that *acr*s are strongly transcribed immediately after phage infection (18), but high levels of *acr* transcription interfere with the transcription of downstream phage genes which are required for the later infection cycle (18,56). We demonstrate that all Aca proteins tested are repressors of their cognate *acr-aca* operons and utilise IR sequences. The use of systems from diverse organisms and mobile elements reinforces the idea the repression mechanism is widespread. Given the potential use of Aca proteins in CRISPR-based gene editing (51), our findings might also extend the toolbox available for such applications.

In conclusion, although the Aca regulatory proteins form distinct families, they all have in common that they bind to different target motifs in their promoters to repress Acr production. Our data suggests the broad importance of *acr* regulation. A possible reason for this is that *acr* expression is toxic, and high expression of Acr by the phage or prophage might negatively affect the viability of the bacterial host or phage.

## DATA AVAILABILITY

Additional data is available at https://github.com/ChrisBrownNZ/Shehreen-2022-Supplement/.

## Supplementary Material

gkac674_Supplemental_Files
