# Supplementary material for: Widespread repression of anti-CRISPR production by anti-CRISPR-associated proteins
Source: Nucleic Acids Res. 2022 Aug 10;50(15):8615–25. doi: 10.1093/nar/gkac674 (PMC9410906; doi:10.1093/nar/gkac674)
Supplement: gkac674_Supplemental_Files [file gkac674_supplemental_files.zip › Shehreen_2022_Supplementary_files.pdf]

## Supplementary Text

## Characteristic features of Aca/AcrIIA1 families and their putative promoters

### Aca1

Aca1-encoding genes were detected downstream of I-E and I-F inhibitors in several *Pseudomonas aeruginosa* Mu-like phages (1-3). This family presents sequence similarities to HTH DNA-binding domain models HTH_24, HTH_XRE, HTH_31 and HTH_8 (4). Homology searching PDB databases found that the protein shares structural similarity with the putative DNA-binding protein ne047 (27%), which belongs to the superfamily containing lambda repressor-like domains [Table 1]. Although we found orthologs in *Acidithiobacillales*, *Rhodanobacter*, *Burkholderia*, and *Achromobacter* (protein size range: 67-89 amino acid), these are rare. The majority of Aca1 homologs belong to various species of *Pseudomonas* [Supplementary data]. Predicted homologs are associated with several I-E (*acrIE1*, *acrIE2*, *acrIE3*, *acrIE4*, *acrIE4-F7*, *acrIE5*), I-F (*acrIF1*, *acrIF2*, *acrIF3*, *acrIF4*, *acrIF5*, *acrIF6*, *acrIF7*, *acrIF11*, *acrIF15*), or I-C *acr*s (*acrIC3*, *acrIC4*, *acrIC5*) and a candidate *acr* c151010 (5) [Figure 2B]. Certain large homologs (protein size range: 100-195 amino acids) are not associated with known *acr* genes. These were found in *Neisseria*, *Moraxella*, *Oceanococcus*. A previous study on related *Pseudomonas* phages demonstrated Aca1 can auto-regulate the *acr-aca1* promoter by binding to inverted repeats located between the -35 and -10 sequences (6). Corroborating these findings, similar inverted repeats (aggaACa($N_0$)tgTtcCt, 'N' denotes the number of spacing nucleotides between the two half-sites, conserved nucleotides are written in upper case) were found upstream of -35 and between predicted -35 and -10 sequences in the high confidence training dataset (n=27) [Figure S4]. The motif was then searched for in all 241 (Refseq_bacteria, n=93; Refseq_Plasmid, n=11, and IMG/VR, n=137) putative promoters having *aca1* in an operon. About half (51.4%,124/241) of the sequences analysed exhibit similar motifs that were discovered from the high confidence training dataset. Although the motif is present in the majority of the *acr-aca1* operons, it was more frequently absent in cases where *aca1* was associated with certain putative *acr* genes or occurred standalone.

**Aca2**

Aca2 has been found in diverse Gammaproteobacteria in an operon with various I-F anti-CRISPRs (7). Later, Aca2 was found to be associated with *acrIIC1* (a Cas9 inhibitor) of the *Brackiella oedipodis* integrated conjugative element (7). The protein has a pfam HTH domain named DUF1870 and is relatively larger than other Aca proteins (for example, Aca2 in *Oceanimonas smirnovii* is 125 amino acids long) [Figure 3]. It shares structural similarity with the YdiL family protein containing lambda repressor like DNA binding domains (24%) [Table 1] which is corroborated by a recent study on the structure of Aca2 (8,9). An HMM was constructed with 25 Aca2 proteins to identify orthologs among bacteria. More diverse homologs were identified in *Shigella*, *Escherichia*, *Yersinia*, *Citrobacter*, *Vibrio*, *Enterococcus*, *Streptomyces* phage, *Serratia*, *Proteus*, *Shewanella*, and others [Figure 2A]. The homologs (protein size: 116-151 amino acid) are associated with both type I (*acrIE9*, *acrIF8*, *acrIF9*, *acrIF15*), type II (*acrIIC4* and *acrIIC5*) and candidate *acr* genes (c70537 (predicted target type I-C), c149605 (predicted target type I-B), c192901(*acrIF8* family, associated with type I-F) and c229638 (predicted target type I-E) (5). An imperfect inverted repeat (gGttCGcaAttGcgacc), similar to IR1(10), is found in the trained sequences (n=9, including 2 from (10) [ Figure 4]. The motif lies between the -35 and -10. Additionally, we found the similar motif downstream of -10 sequences (in which case it has a slightly different nucleotide composition than the IR1 region and was hence denoted as IR2 here and in the previous study) [Figure S4] (10). Both of these motifs were analysed in 669 *aca2*-positive sequences (Refseq_bacteria, n=249; Plasmid, n=198; IMG/VR, n=222). Only 48/669 sequences have IR1 and/or IR2 motif. This small proportion is unsurprising, as only 31 out of 669 promoters had a known, or candidate *acr* in the same locus as *aca2*.

**Aca3**

Aca3 was first discovered in the *Neisseria meningitidis*'s integrated MGE/prophage (7). The protein was found in an operon with only one or a group of inhibitors that antagonize the host's CRISPR-Cas type II-C system. Aca3 is predicted to be structurally similar to the transcriptional regulator/antitoxin MqsA (27%) [Table 1]. MqsA autorepresses its own expression by binding to its promoter (11). Similarity searches with the Pfam database showed that the protein may have a HTH_3, HTH_19 or HTH_XRE domain (4) [Table 1]. We found orthologs (protein size range: 67-80 amino acid) among various species of *Neisseria*, *Bibersteinia*, and *Actinobacillus*. They are found in the same operon with genes

encoding the Cas9 inhibitors AcrIIC2, AcrIIC3 and also a candidate Acr c56759 (predicted target type I-C or type I-F) (5). In this present study, a potential binding motif (between -35 and -10) was found from the trained dataset (n=8) [Figure S4 and Figure 4], showing an imperfect inverted repeat (tAcAtcA($N_5$)TGatgta) as previously reported (6). A search based on 372 sequences (Refseq_bacteria, n=249; Plasmid, n= 1; IMG/VR, n=117) found the motif in only 34 instances. The motif was absent in sequences where *aca3* homologs occurred solo.

**Aca4**

Aca4 has been found in a genome of *Pseudomonas aeruginosa*, alongside a gene encoding an AcrIF11 homolog in the same operon (12). A Pfam domain search found that the protein belongs to the HTH_23 and KorA family in the HTH clan and has similarity with TrfB plasmid transcriptional repressor KorA (26%) [Table 1]. KorA is a dimeric repressor that recognizes a palindromic consensus sequence in the operator region and represses the strong *trfA* promoter (13). An HMM search was performed to identify distantly related homologs across bacterial genomes. Only the homologs from different *Pseudomonas* species (protein size range: 65-72 amino acids) [Figure 2] were associated with several type I-C (*acrIC6*, *acrIC7*, *acrIC8*), one type I-E (*acrIE9*), two type I-F (*acrIF11*, *acrIF12*) and the candidate *acr* c24021 (predicted target type III-D, type III-B, type I-F) (5) [Figure 2B]. An imperfect inverted repeat (tacaccc($N_4$)gggtgcA) was discovered in the high confidence training set (n=5) [ Figure 4]. In those sequences, the motif was found twice: between the predicted -35 and -10 region, and downstream of -10 (within the 5′-UTR). The *acr-aca4* promoter sequences (Refseq_bacteria, n= 236; Plasmid, n=25; IMG/VR, n=31) were then searched for the motif. Of these, the motif was found at least once (within 100 bp prior to the *acr* start) in 59 RefSeq bacterial genomes. However, we observed no known *acr-aca4* operon on plasmids. In the IMG/VR database, a similar motif was found in the promoters which have putative *acr* and *aca4* in a single operon.

**Aca5**

Aca5 was initially found in an operon containing *acrIF11* homologs in a *Pectobacterium carotovorum* genome (12). Yin *et al.* predicted two different domains in different homologs (HTH_3 and HTH_28) (4). The protein was searched against PDB. The protein has structural similarity to the Rep-Ant (repressor-antirepressor) complex from a *Salmonella* temperate phage (26%) [Table 1]. In this study, an HMM search predicted

homologs (protein size range: 51-88 amino acid) within the genera *Citrobacter, Dickeya*, *Klebsiella*, *Serratia*, *Pectobacterium*, *Photorhabdus*, and *Escherichia* [Figure 2A]. These were associated with a type I-E inhibitor (*acrIE8*), eight I-F inhibitors (*acrIF15*, *acrIF16*, *acrIF17*, acrIF18, *acrIF19*, *acrIF20*, *acrIF21*, and *acrIF22*) and candidate *acr* c51174 [Figure 2D]. This candidate *acr* was found in genomes with CRISPR-Cas subtype I-F and I-C self-targeting (5). The putative promoters were extracted from *acr-aca5* operons and common motifs were identified by MEME (n=13). A nearly perfect inverted repeat (ATAatCaa ($N_3$)TtGatTAT) [Figure 4] was discovered between the predicted -35 and -10 promoter motifs. A similar motif (IR2) was also found upstream of the -35 regions in 6 out of 15 sequences [Figure S4]. The motif was searched against 164 promoter sequences (Refseq_bacteria, n= 98; Plasmid, n=7; IMG/VR, n=59) associated with *aca5* genes. Almost all promoters containing known and candidate *acr* genes have the binding motif (in some cases multiple times within 100 bp before the *acr* initiation codon). However, motifs were not frequently found when the *aca5* homologs occurred standalone (no known or putative *acr* genes in the operon).

**Aca6**

Aca6 was discovered in the marine bacterium *Alcanivorax* sp., which degrades alkane, and is associated with AcrIF11 homologs (12). The protein has an HTH_3 domain (4) and shares structural similarity with the C-terminal domain of *Vibrio cholerae* HigA2 antitoxin (24%) [Table 1]. The HMM search predicts 128 proteins from diverse species such as *Janthinobacterium*, *Chitinimonas*, *Comamonas*, *Alcanivorax*, *Acidovorax*, *Achromobacter*, *Xanthomonas*, *Vulcaniibacterium*, and *Oceanospirillaceae* but only eight of them (protein size: 67-80 amino acid) were associated with *acrIC6*, candidate *acr* c51174 and c22766 [Figure 2]. These clusters were found in bacterial genomes with CRISPR-Cas subtype I-F and I-C (5). There were 266 promoter sequences (Refseq_bacteria, n= 239; Plasmid, n=6; IMG/VR, n=21) associated with *aca6* homologs. However, only four of them had *aca6* occur with either a known or candidate *acr*. Only one had an IR (atgcGCAC($N_3$)gTGCgcttt) between -35 and -10 [Figure 4 and Figure S4].

**Aca7**

Aca7 was found with AcrIF11 homologs in the halophilic bacterium *Halomonas caseinilytica* (12). Similar to Aca6, it has an HTH_3 domain (4), but shares 24% structural similarity with the Rep-Ant complex from a *Salmonella* temperate phage and 20% to

regulatory protein C [Table 1]. This C-protein regulates the restriction-modification system Kpn2I. The C protein binds upstream of the strong methyltransferase promoter and inhibits the gene transcription by preventing the interaction of the RNA polymerase sigma subunit with -35 sequence (14). We identified diverse homologs (Protein size: 67-71 amino acid) of Aca7 from different species of *Halomonas* and *Kushneria* are associated only with *acrIF11* or candidate *acr* c10901 [Figure 2] (5). Homologs were also found in the *Alteromonas* and *Idiomarina* genomes. None of these Aca7 homologs was associated with a known/candidate *acr*. The putative promoters (n=3) were extracted from the *acr-aca7* operons and common motifs were identified by MEME. An imperfect inverted repeat (TGatAACT($N_1$)AGTTatCa) was discovered between the predicted -35 and -10 promoter motifs. Next, the motif was searched against 205 promoter sequences (Refseq_bacteria, n= 18; Plasmid, n=8; IMG/VR, n=179) associated with *aca7* genes. Although Aca7 homologs are found encoded in plasmids, none of these are associated with Acrs. In addition, none of the putative promoters in the plasmids contained the predicted motif. Also, in IMG/VR, none of the *aca7* homologs are associated with known *acr* genes, but 17 of 179 analysed sequences have the motif within 400 bp upstream of the initiation codon of the next gene.

**Aca9**

The *aca9* gene was found in an operon containing *acrIF22* in a plasmid of *Klebsiella pneumoniae* (15). The HTH domain was predicted by searching against all Pfam sequences. Similar to Aca6, Aca7 and AcrIIA1, Aca9 has an HTH_3 DNA binding motif. The Aca9 protein shares structural similarity with the C-terminal domain of *Vibrio cholerae* HigA2 antitoxin (23%) [Table 1], a modular protein with a C-terminal helix-turn-helix domain for dimerization and a DNA binding domain (16). Orthologs of Aca9 have been found in several species, particularly in plasmids and prophages. Consistent with our previous study (15), the HMM search found Aca9 homologs in different species of *Raoultella*, *Serratia*, *Klebsiella*, *Pectobacterium*, *Aeromonas*, *Enterobacteriaceae*, *Shigella*, *Chryseobacterium*, and *Flavobacterium* [Supplementary data and Figure 2]. The predicted homologs (protein size range: 69-76 amino acid) are associated with *acrIF22*, *acrIF17*, and a candidate *acr* c51174 [Figure 2] (5). There were 572 promoter sequences (Refseq_bacteria, n= 458; Plasmid, n=57; IMG/VR, n=57) associated with *aca9* homologs. Only three of them had a known *acr* in an operon with *aca9*. A conserved inverted repeat termed IR1 (caccTgn($N_1$)CaGgTg) was predicted between the -35 and -10

region in three sequences which were associated with a known *acr* . A similar motif (IR2) was found upstream of the -35 site in all three instances. The motif was then searched against the large (n=572) set. The motif was ubiquitously found within 400 bp.

**Aca10**

Aca10 has recently been reported in *Pseudomonas citronellolis* and is encoded adjacent to *acrIC7* and *acrIC6* (17). Similar to Aca1, Aca10 has an HTH_31 domain. The protein shares structural similarity with the HigBA2 toxin-antitoxin complex from plasmid Rts1 (30%), particularly with the antitoxin module [Table 1]. A broad range of Aca10 homologs (size range: 62-74 amino acid) was identified across diverse bacteria, e.g. *Cupriavidus*, *Burkholderia*, *E. coli*, *Acetobacter*, *Pseudomonas*, *Alcanivorax*, *Methylobacter*, *Rahnella*, etc. [Supplementary data and Figure 2]. These proteins are associated with type I-C inhibitors (*acrIC6* and *acrIC7*), type I-F inhibitors (*acrIF16* and *acrIF17*) and a candidate *acr* c56759 [Figure 2]. The candidate *acr* was predicted to target type I-C and type I-F (5). The putative promoters (n=3) were extracted from *acr-aca10* operons and common motifs were identified. An imperfect inverted repeat (atacgcTC($N_3$)GAgCGtAt) was discovered between the predicted -35 and -10 regions. In addition to one between -35 and -10, in one instance, the motif was found upstream of -35 sequences. Then the motif was searched against 221 promoter sequences associated with *aca10* genes (Refseq_bacteria, n= 146; Plasmid, n=75). Since Aca10 was reported very recently (17), homologs were found to be associated with putative *acr* genes. Around 38.5% (85/221) of analysed sequences have the predicted motif.

**AcrIIA1**

AcrIIA1 is a Cas9 inhibitor encoded by *Listeria monocytogenes* prophages (18). The N-terminal domain of AcrIIA1 contains either an HTH_26 or an HTH_3 domain (4) which binds and auto-regulates operon expression and the C-terminal domain acts as an Acr (19,20). Homologs can be found in *Firmicutes* MGEs and chromosomes (18). The protein has structural similarity with prophage lp1 protein 11 (26%) and *Pseudomonas putida* antitoxin GraA (21%) [Table 1]. The antitoxin GraA dimerizes, binds cooperatively at opposite sides of the operator sequence and autorepresses the *graTA* promoter (21). An HMM search of the NCBI-NR database revealed that homologs (protein size range: 75-174 amino acids) are widespread among *Oceanospirillum multiglobuliferum*, *Enterococcus* and different species of *Listeria*. Small representatives only contain the HTH

domain, but not the C-terminal *acr* domain. The putative promoters (n=22) were extracted from *acrIIA1*-containing operons and common motifs were identified. A nearly perfect inverted repeat (TACTACGa($N_4$)TCGTAGTA) was discovered between the predicted -35 and -10 like promoter elements. The motif was searched against 675 promoter sequences (Refseq_bacteria, n= 444; Plasmid, n=2; IMG/VR, n=229). The binding motif was found in 458/675 putative promoters.

## Supplementary Tables and Figures

**Supplementary Table S1: Chosen parameters for psi-blast**

| Aca/AcrIIA1 | Query cover (%) | Amino acid identity (%) | e-value | No. of homologues selected for HMM after 4-iterations |
|---|---|---|---|---|
| Aca1 | >80 | >50 | $<10^{-18}$ | 191 |
| Aca2 | >80 | >50 | $<10^{-8}$ | 25 |
| Aca3 | >85 | >50 | $<10^{-19}$ | 5 |
| Aca4 | >93 | >61 | $<10^{-27}$ | 10 |
| Aca5 | >92 | >50 | $<10^{-16}$ | 8 |
| Aca6 | >88 | >50 | $<10^{-16}$ | 9 |
| Aca7 | >93 | >50 | $<10^{-21}$ | 5 |
| Aca8 | >91 | >45 | $<10^{-16}$ | 5 |
| Aca9 | >80 | >35 | $<10^{-10}$ | 15 |
| Aca10 | >80 | >68 | $<10^{-24}$ | 12 |
| AcrIIA1 | 100% | >80 | $<10^{-87}$ | 6 |

**Supplementary Table S2: Number of Aca-like proteins analysed, those with >40% query coverage from Figure S1)**

| Aca/AcrIIA1 | Acr(+) | Acr(-) |
|---|---|---|
| Aca1 | 90 | 10 |
| Aca2 | 35 | 38 |
| Aca3 | 21 | 15 |
| Aca4 | 17 | 56 |
| Aca5 | 27 | 12 |
| Aca6 | 8 | 67 |
| Aca7 | 6 | 2 |
| Aca9 | 7 | 10 |
| Aca10 | 12 | 69 |
| AcrIIA1 | 163 | 3 |

**Supplementary Table S3: Number of promoter sequences for the analyses**

| | Aca1 | Aca2 | Aca3 | Aca4 | Aca5 | Aca6 | Aca7 | Aca9 | Aca10 | AcrIIA1 |
|---|---|---|---|---|---|---|---|---|---|---|
| **Promoters** | 93 | 249 | 254 | 236 | 98 | 239 | 15 | 458 | 146 | 444 |
| **High confidence sets** | 27 | 9 | 9 | 6 | 15 | 4 | 4 | 3 | 3 | 25 |
| **High confidence sets with DNA motifs** | 27 | 8 | 8 | 5 | 13 | 2 | 3 | 3 | 3 | 22 |

**Supplementary Table S4: List of oligonucleotides used in this study**

| Name | Sequence | Description | Restriction site |
|---|---|---|---|
| pGR2_F | GTCCACACAATCTGCCCTTT | Forward primer for screening/sequencing pGR2-derived plasmids | |
| pGR2_R | TCTTCGGAGGAAGCCATCTA | Reverse primer for screening/sequencing pGR2-derived plasmids | |
| pCDF-1b_F | TAATACGACTCACTATAGGG | Forward primer for screening/sequencing pCDF-1b-derived plasmids | |
| pCDF-1b_R | GCTAGTTATTGCTCAGCGG | Reverse primer for screening/sequencing pCDF-1b-derived plasmids | |
| Aca2_scPromoter_F_spe1 | TTTTACTAGTTATTGTGGCGCTGTGTGATTTAC | Forward primer to clone *aca2* wild type and scrambled promoter in pGR2 | SpeI |
| Aca2_scPromoter_R_pst1 | TTTTCTGCAGTGTGGAATCCTCGTTAGGAG | Reverse primer to clone *aca2* wild type and scrambled promoter in pGR2 | PstI |
| Aca1_F_NcoI | TTTTCCATGGAGGAGGACACGGGTGAAACCTG | Forward primer to clone *aca1* gene in pCDF-1b | NcoI |
| Aca1_R_HindIII | TTTTAAGCTTCTAGCTCTCGCTAGCCAGG | Reverse primer to clone *aca1* gene in pCDF-1b | HindIII |
| Aca2_F_NcoI | TTTTCCATGGAGGAGGACACGGATGACAAACAAAG | Forward primer to clone *aca2* gene in pCDF-1b | NcoI |

| | | | |
|---|---|---|---|
| Aca2_R_HindIII | TTTTAAGCTTTTAGATTAAATCCGCGTGACCCTCAG | Reverse primer to clone *aca2* gene in pCDF-1b | HindIII |
| Aca3_F_NcoI | TTTTCCATGGAGGAGGACACGGATGATTGACAG | Forward primer to clone *aca3* gene in pCDF-1b | NcoI |
| Aca3_R_HindIII | TTTTAAGCTTTTATAGCTTCCTCACATATTCCAGC | Reverse primer to clone *aca3* gene in pCDF-1b | HindIII |
| Aca4_F_NcoI | TTTTCCATGGAGGAGGACACGGATGACGGAAGAG | Forward primer to clone *aca4* gene in pCDF-1b | NcoI |
| Aca4_R_HindIII | TTTTAAGCTTTCAGGTAAATACCCGCTTGGC | Reverse primer to clone *aca4* gene in pCDF-1b | HindIII |
| Aca5_F_NcoI | TTTTCCATGGAGGAGGACACGGATGCGACTAAC | Forward primer to clone *aca5* gene in pCDF-1b | NcoI |
| Aca5_R_HindIII | TTTTAAGCTTTTAGAGTGGCGGGATTTCTCG | Reverse primer to clone *aca5* gene in pCDF-1b | HindIII |
| Aca6_F_NcoI | TTTTCCATGGAGGAGGACACGGATGGACAAGAATG | Forward primer to clone *aca6* gene in pCDF-1b | NcoI |
| Aca6_R_HindIII | TTTTAAGCTTTCAGTCTTGGTAGCCGAGC | Reverse primer to clone *aca6* gene in pCDF-1b | HindIII |
| Aca7_F_NcoI | TTTTCCATGGAGGAGGACACGGATGATCGAC | Forward primer to clone *aca7* gene in pCDF-1b | NcoI |
| Aca7_R_HindIII | TTTTAAGCTTTCAGTTTTCGCCACCTTGG | Reverse primer to clone *aca7* gene in pCDF-1b | HindIII |

| | | | |
|---|---|---|---|
| Aca9_F_NcoI | TTTTCCATGGAGGAGGACACGGATGAAAAACAC | Forward primer to clone *aca9* gene in pCDF-1b | NcoI |
| Aca9_R_HindIII | TTTTAAGCTTTCATTCAGATCTGCTCATCCACTC | Reverse primer to clone *aca9* gene in pCDF-1b | HindIII |
| Aca10_F_NcoI | TTTTCCATGGAGGAGGACACGGATGAGC | Forward primer to clone *aca10* gene in pCDF-1b | NcoI |
| Aca10_R_HindIII | TTTTAAGCTTTCACGAACTGGGGCTGG | Reverse primer to clone *aca10* gene in pCDF-1b | HindIII |
| AcrIIA1_F_NcoI | TTTTCCATGGAGGAGGACACGGATGTC | Forward primer to clone *acrIIA1* gene in pCDF-1b | NcoI |
| AcrIIA1_R_HindIII | TTTTAAGCTTTTATATCAATTCGTTTTTCTTCTCTTTTAA | Reverse primer to clone *acrIIA1* gene in pCDF-1b | HindIII |
| Promoter_Aca1 | tcgtcttcacctcgagaaatc**ACTAGT**AGCCTGCGTAGCAGAAAACCCGACAAAAGGAACAATGTTCCTATTTACACAGAGGAACAATGTTCCTATTATATGAGCCATGCCAGCCGCAACGGCGAGGCACAGCAAGGAGCGAGGCCAATGCAT**CTGCAG**cctgttgatagatccagtaatgac | gBlock template for cloning of wt-IR *acrIE1-acrIF3-acrIF5-aca1* promoter insert | SpeI, PstI |
| Sc_Promoter_Aca1 | tcgtcttcacctcgagaaatc**ACTAGT**AGCCTGCGTAGCAGAAAACCCGACAAAAGCATGAATCTTCCTATTTACACAGAGCATGAATCTTCCTATTATATGAGCCATGCCAGCCGCAACGGCGAGGCACAGCAAGGAGCGAGGCCAATGCAT**CTGCAG**cctgttgatagatccagtaatgac | gBlock template for cloning of sc-IR *acrIE1-acrIF3-acrIF5-aca1* promoter insert | SpeI, PstI |
| Promoter_Aca3 | tcgtcttcacctcgagaaatc**ACTAGT**TTTTGTTTAAAAAAGTGCTTGTGATACATCATTTAATGATGTAATATACACACATGGACAGACAACAAGCCTGCCACCGACACCTTGACGGATTCAA | gBlock template for cloning of wt-IR *acrIIC3-acrIIC2-aca3* promoter insert | SpeI, PstI |

| | | | |
|---|---|---|---|
| | GGATAAACGAAAGGATTTCAAAAATGCAT**CTGCAG**cctgttgatagatccagtaatgac | | |
| Sc_Promoter_Aca3 | tcgtcttcacctcgagaaatc**ACTAGT**TTTTGTTTAAAAAAGTGCTTGTGATTCTTCTTTTAATCATGTAATATACACACATGGACAGACAACAAGCCTGCCACCGACACCTTGACGGATTCAAGGATAAACGAAAGGATTTCAAAAATGCAT**CTGCAG**cctgtgatagatccagtaatgac | gBlock template for cloning of sc-IR *acrIIC3-acrIIC2-aca3* promoter insert | SpeI, PstI |
| Promoter_Aca4 | tcgtcttcacctcgagaaatc**ACTAGT**GTGGGAGATAGAGCATCTGCCTGCCCAATCAGCCTGCTTGAACATATCCCTTTAAAGGGATAACCTCAAACCCAACGTTGGGCTATCAACCCAACCTCCAACGCCCCTGCCGAGAAAGACATGCAT**CTGCAG**cctgttgatagatccagtaatgac | gBlock template for cloning of wt-IR *acrIF12-aca4* promoter insert | SpeI, PstI |
| Sc_Promoter_Aca4 | tcgtcttcacctcgagaaatc**ACTAGT**GTGGGAGATAGAGCATCTGCCTGCCCAATCAGCCTGCTTGAACATATCGCTGTAAAGTGAAAACCTCAAACCCAACGTTGGGCTATCAACCCAACCTCCAACGCCCCTGCCGAGAAAGACATGCAT**CTGCAG**cctgttgatagatccagtaatgac | gBlock template for cloning of sc-IR *acrIF12-aca4* promoter insert | SpeI, PstI |
| Promoter_Aca5 | tcgtcttcacctcgagaaatc**ACTAGT**AGCCTCACCTCCCCTGTGGGGTTGGCGCCGTTTGTGATTGAAAAAGGTAAAATAAGTGTTGCTACATAGTCAGTTTTTGACTATATTATTTATATCGAAACGAGATTGAGGGATTCACAAATGCAT**CTGCAG**cctgttgatagatccagtaatgac | gBlock template for cloning of wt-IR *acrIF20-acrIF19-acrIF17-acrIF16-aca5* promoter insert | SpeI, PstI |
| Sc_Promoter_Aca5 | tcgtcttcacctcgagaaatc**ACTAGT**AGCCTCACCTCCCCTGTGGGGTTGGCGCCGTTTGTGATTGAAAAAGGTAAAATAAGTGTTGCTACTAAGTGAGTTTTTCACTATATTATTTATATCGA | gBlock template for cloning of sc-IR *acrIF20-acrIF19-acrIF17-acrIF16-aca5* promoter insert | SpeI, PstI |

| | | | |
|---|---|---|---|
| | AACGAGATTGAGGGATTCACAAATGCAT**CTGCAG**cctgttg atagatccagtaatgac | | |
| Promoter_Aca6 | tcgtcttcacctcgagaaatc**ACTAGT**AAATCTCGGTGGTAGAAAT AGCTTGCGAATGTGCAAACTTTGCACTACAGTGGAAAC CATGGACAGCAACCCTGCACATCCGACCAGGCGGACCC TGGAATCCTCTGAAGGAACACATCAATGCAT**CTGCAG**cc tgttgatagatccagtaatgac | gBlock template for cloning of wt-IR *candidate-acrIC6-aca6-putative* promoter insert | SpeI, PstI |
| Sc_Promoter_Aca6 | tcgtcttcacctcgagaaatc**ACTAGT**AAATCTCGGTGGTAGAAAT AGCTTGCGAATGTCGTAACTTTGCACTACAGTGGAAAC CATGGACAGCAACCCTGCACATCCGACCAGGCGGACCC TGGAATCCTCTGAAGGAACACATCAATGCAT**CTGCAG**cc tgttgatagatccagtaatgac | gBlock template for cloning of sc-IR *candidate-acrIC6-aca6-putative* promoter insert | SpeI, PstI |
| Promoter_Aca7 | tcgtcttcacctcgagaaatc**ACTAGT**TCGCGCCGTCAGGTGGGGA GCATCGTCGGGCATGGGTCCACCGCATTCATTGGCCCTG CCGCATTTGACATGATAACTCAGTTATCATAAACTGAAT TCAACAGATGAGGAGACGGCGCCATGCAT**CTGCAG**cctgt tgatagatccagtaatgac | gBlock template for cloning of wt-IR *acrIF11-aca7-putative* promoter insert | SpeI, PstI |
| Sc_Promoter_Aca7 | tcgtcttcacctcgagaaatc**ACTAGT**TCGCGCCGTCAGGTGGGGA GCATCGTCGGGCATGGGTCCACCGCATTCATTGGCCCTG CCGCATTTGACAACATATCTCACTAATCATAAACTGAAT TCAACAGATGAGGAGACGGCGCCATGCAT**CTGCAG**cctgt tgatagatccagtaatgac | gBlock template for cloning of sc-IR *acrIF11-aca7-putative* promoter insert | SpeI, PstI |
| Promoter_Aca9 | tcgtcttcacctcgagaaatc**ACTAGT**TGTGCAAAGCCCCAAACAA AATAACACCTGACAGGTGATAAATACATTGTGAAACAC ACCTGTCAGGTGTACACTGAATTCATCGAAAGCAATAC | gBlock template for cloning of wt-IR *acrIF22-aca9* promoter insert | SpeI, PstI |

| | | | |
|---|---|---|---|
| | TGCTTTCCCAGTGGAGGATGTAAAAATGCAT**CTGCAG**cc tgttgatagatccagtaatgac | | |
| Sc_Promoter_Aca9 | tcgtcttcacctcgagaaatc**ACTAGT**TGTGCAAAGCCCCAAACAA AATAACACCTGACAGGTGATAAATACATTGTGAAACTC ACCAGTGACGAGTACACTGAATTCATCGAAAGCAATAC TGCTTTCCCAGTGGAGGATGTAAAAATGCAT**CTGCAG**cc tgttgatagatccagtaatgac | gBlock template for cloning of sc-IR *acrIF22-aca9* promoter insert | SpeI, PstI |
| Promoter_Aca10 | tcgtcttcacctcgagaaatc**ACTAGT**CATGTGGTCCTCGCAAAAAG ATTTGCAAATACGCTCATTGAGCGTATTCTACACCCATG GCAGGCGTAAACGCCGACCACCGCCCCGGCGGAACCGG GCTTCCTGATAGGAGCAACATCAATGCAT**CTGCAG**cctgtt gatagatccagtaatgac | gBlock template for cloning of wt-IR *acrIC7-acrIC6-aca10* promoter insert | SpeI, PstI |
| Sc_Promoter_Aca10 | tcgtcttcacctcgagaaatc**ACTAGT**CATGTGGTCCTCGCAAAAAG ATTTGCAAATACGCAGTTTGAGGCTTTTCTACACCCATG GCAGGCGTAAACGCCGACCACCGCCCCGGCGGAACCGG GCTTCCTGATAGGAGCAACATCAATGCAT**CTGCAG**cctgtt gatagatccagtaatgac | gBlock template for cloning of sc-IR *acrIC7-acrIC6-aca10* promoter insert | SpeI, PstI |
| Promoter_AcrIIA1 | tcgtcttcacctcgagaaatc**ACTAGT**AGACTTCGGTTTGATGCTTT TTTTATTTTAAAATAAATTTAATAAAATTATTGACTACT ACGAATAATCGTAGTATAATGTAAATATAGTAAACAAA CCAACTAAAAAGGATGATGAAAAATGCAT**CTGCAG**cctgt tgatagatccagtaatgac | gBlock template for cloning of wt-IR *acrIIA4-acrIIA1* promoter insert | SpeI, PstI |
| Sc_Promoter_AcrIIA1 | tcgtcttcacctcgagaaatc**ACTAGT**AGACTTCGGTTTGATGCTTT TTTTATTTTAAAATAAATTTAATAAAATTATTGACAACA ACCAATAAACCTAGTATAATGTAAATATAGTAAACAAA | gBlock template for cloning of sc-IR *acrIIA4-acrIIA1* promoter insert | SpeI, PstI |

| | | | |
|---|---|---|---|
| | CCAACTAAAAAGGATGATGAAAAATGCAT**CTGCAG**cctgttgatagatccagtaatgac | | |
| Wt-Aca1 | tcgtcttcacctcgagaaatcgaattcttgacaattaatcatccggctcgtataatgtgtggaattgtgagcggataacaatttcacacaggaaacagaccgagctcaggaggacacggGTGAAACCTGACGCCTCCAACCACAATCCAGACCCGCGCTACCTGCGCGGGCTGTACGAGCGAGCCGGCCTGAAGCAGGAAGAAGCTGCCAGGCGGATCGGAATCACCGCCCGCGCGCTGAGGAATTACGTCAGCGAAACCGCCGGCAGAGAGGCGCCGTATCCCGTTCAATTCGCGCTTGAGTGCCTGGCTAGCGAGAGCTAGgcatgcaagcttcctgttgatagatccagtaatgac | gBlock template for cloning of *aca1* | |
| Wt-Aca3 | tcgtcttcacctcgagaaatcgaattcttgacaattaatcatccggctcgtataatgtgtggaattgtgagcggataacaatttcacacaggaaacagaccgagctcaggaggacacggATGATTGACAGGCCCGAATTGGGATACACGCCCGCCAATTTGAAGGCAGTGCGGCAAAAGTACGGGCTGACGCAAAACCAGGTTGCCGATATTACTGGCACAACCTTGTCGACAGCCCAAAAATGGGAGGCGGCGATGAGTTTGAAAACTCATTCCGATATGCCTCATACGCGCTGGCTGATTTTGCTGGAATATGTGAGGAAGCTATAAgcatgcaagcttcctgttgatagatccagtaatgac | gBlock template for cloning of *aca3* | |
| Wt-Aca4 | tcgtcttcacctcgagaaatcgaattcttgacaattaatcatccggctcgtataatgtgtggaattgtgagcggataacaatttcacacaggaaacagaccgagctcaggaggacacggATGACGGAAGAGCAGTTTTCAGCCCTTGCTGAGTTGATGCGCCTGCGCGGAGGTCCTGGCGAGGATGCTGCCCGACTGGTGTTGGTAAATGGTCTCAAGCCTACCGATGCTGCCCGGAAGACAGGTATCACTCCCCAAGCCGTGAATAAGACCCTCAGCAGTTGCCGGCGTGGCATTGAACTTGCCAAGCGGGTATTTACCTGAgcatgcaagcttcctgttgatagatccagtaatgac | gBlock template for cloning of *aca4* | |

| | | | |
|---|---|---|---|
| Wt-Aca5 | tcgtcttcacctcgagaaatcgaattcttgacaattaatcatccggctcgtataatgtgtggaatt gtgagcggataacaatttcacacaggaaacagaccgagctcaggaggacacggATGC GACTAACTGAATTCATCAATCAACACTTCGACGGCAAT AAAGCGGCATTCGCTCGGCACATGGGCGTTGATGCGCA GGCCGTTAACAAATGGATTAAATCAGAGTGGTTTGTCA GCACTACTGATGACAATAAAATCTATCTCAGCTCTGTGC GTCGAGAAATCCCGCCACTCTAAgcatgcaagcttcctgttgatagatc cagtaatgac | gBlock template for cloning of *aca5* | |
| Wt-Aca6 | tcgtcttcacctcgagaaatcgaattcttgacaattaatcatccggctcgtataatgtgtggaatt gtgagcggataacaatttcacacaggaaacagaccgagctcaggaggacacgGATGG ACAAGAATGATTTTGTGCAGTGGCACAAGCGCCTGGGC TTTGCCTCTCAATCAGAAGGCGCCGCGGCGCTCGGCGT CAAGCGCAGCACCTACGCCAACTACATGGGCGGCATAT CGCGTACCACGGGCAAGCCGGTGGATTACGACCTGCGG CTGGCCTACGCCTGCGCCGCCATTGAGGCGGGCATCAA GCCGCTCGGCTACCAAGACTGAgcatgcaagcttcctgttgatagatcc agtaatgac | gBlock template for cloning of *aca6* | |
| Wt-Aca7 | tcgtcttcacctcgagaaatcgaattcttgacaattaatcatccggctcgtataatgtgtggaatt gtgagcggataacaatttcacacaggaaacagaccgagctcaggaggacacggATGA TCGACGCTCGAAAGTACTACAATCCTGACCTAGCGCCT GAGTTGGTTAGTCGCGCCCTCGCCGTTACTGGGACACA GAAGGAGCTTGCCGAGCGGCTCGACGTATCCCGCATCT ACATCCAGTTGCTTGGCAAGGGGCAGAAGACGATGTCA TATGCTGTGCAAGTGATGCTTGAGCAGGTGATCCAAGG TGGCGAAAACTGAgcatgcaagcttcctgttgatagatccagtaatgac | gBlock template for cloning of *aca7* | |
| Wt-Aca9 | tcgtcttcacctcgagaaatcgaattcttgacaattaatcatccggctcgtataatgtgtggaatt gtgagcggataacaatttcacacaggaaacagaccgagctcaggaggacacggATGA AAAACACACGACTGGTAGATGCCCAGAGCAAACTCGGC | gBlock template for cloning of *aca9* | |

| | | | |
|---|---|---|---|
| | TTCTCCAAGGCTGAAATGGCCCGCGCACTCTCTGTTCATTACAACACTTACGATAAGTGGGAACGCGGAGAGCAAAAGCCTCAGGCAGCCGTTTACACAGCCGTGGACATGCTTCTGTTCATGCACGCTAAAGGCATTCTTACCGAGTGGATGAGCAGATCTGAATGAgcatgcaagcttcctgttgatagatccagtaatgac | | |
| Wt-Aca10 | tcgtcttcacctcgagaaatcgaattcttgacaattaatcatccggctcgtataatgtgtggaattgtgagcggataacaatttcacacaggaaacagaccgagctcaggaggacacggATGAGCAGCGCTACTCCTGATCCCGCAGAAATCCTCACCGCCCGCAAGGCGGTCGGCCTATCTCAGACGGCAGCCGCTGCGTTGGTGCACAGCAGCCTGCGAACCTGGCAGCAGTGGGAGGCCGGAGACAGGCGTATGCACCCCGGCCTGTGGGAACTATTCCTGCTGAAGACTCAGTTGCCCAGCCCCAGTTCGTGAgcatgcaagcttcctgttgatagatccagtaatgac | gBlock template for cloning of *aca10* | |
| Wt-AcrIIA1 | tcgtcttcacctcgagaaatcgaattcttgacaattaatcatccggctcgtataatgtgtggaattgtgagcggataacaatttcacacaggaaacagaccgagctcaggaggacacggATGTCAATAAAACTATTAGATGAATTCTTAAAAAAACACAATAAAACGAGGTATCAGTTAAGCAAACTGACTGGTATCTCGCAAAACACATTGAACGATTACAATAAAAAAGAGTTAAACAAGTATTCTGTTTCATTCTTGCGCGCACTCTCAATGTGTGCAGGAATATCTACATTTGATGTTTTCAACGAACTAGAAGAATTAGAAAAAAACTATGATGATCTCGCAGGATTTAAGCACTTGTTAGATAAGTATAAGTTGTCATTTTCAGCGCAAGAATTCGAATTATACTGCTTAATCAAAGAGTTTGAATCTGCGAACATTGAAGTGCTCCCTTTTACATTTAATAGATTCGAAAATGAAACGCATGTAGATATAGAAAAAGATGTTCGAAAAGCACTGGAAAATGCTATCACTGTGTTAAAAGAGAAGAAAAACGAATTGATATAAgcatgcaagcttcctgttgatagatccagtaatgac | gBlock template for cloning of *acrIIA1* | |

## Supplementary Table S5: Bacterial strains used in this study

| Name | Genotype/Phenotype | Reference |
|---|---|---|
| *Escherichia coli* | | |
| **DH5α** | $F^-$ , ϕ80Δd*lac*ZM15, Δ(*lac*ZYA–*arg*F)U169, *end*A1, *rec*A1, *hsd*R17 ($r_K$ - $m_K$ + ), *deo*R, t*hi*-1, *sup*E44, λ- , *gyr*A96, *rel*A1 | (22) |
| **BL21(DE3)** | Str., B, F–, *omp*T, *gal*, *dcm*, *lon*, hsd$S_B$($r_B$ – $m_B$ – ), λ(DE3, [*lac*I, *lac*UV5-*T7p07*, *ind*1, *sam*7, *nin*5]), [$malB^+$]$_{K\text{-}12}$($\lambda^S$) | (23) |

## Supplementary Table S6: Plasmids used in this study

| Plasmid Name | Backbone | Description | Reference |
|---|---|---|---|
| pGR2 | pGR and pHERD30T | *rfp* reporter plasmid for insertion of *acr-aca* promoters, arabinose-inducible *gfp* reporter, GmR, pBR322 origin | unpublished |
| pCDF-1b | | IPTG-inducible T7 lac promoter containing vector, SmR, CloDF13 origin | Novagen |
| pSS22 | pCDF-1b | PCR amplification of *aca1* gene from gBlock and restriction cloning of *aca1* gene into pCDF-1b vector using NcoI and HindIII | This study |
| pPF1532 | pBAD30 | Template for amplification of *aca2* | (10) |
| pSS23 | pCDF-1b | PCR amplification of *aca2* gene from pPF1532 and restriction cloning of *aca2* gene from into pCDF-1b vector using NcoI and HindIII | This study (10) |
| pSS24 | pCDF-1b | PCR amplification of *aca3* gene from gBlock and restriction cloning of *aca3* gene into pCDF-1b vector using NcoI and HindIII | This study |
| pSS25 | pCDF-1b | PCR amplification of *aca4* gene from gBlock and restriction cloning of *aca4* gene into pCDF-1b vector using NcoI and HindIII | This study |
| pSS26 | pCDF-1b | PCR amplification of *aca5* gene from gBlock and restriction cloning of *aca5* gene into pCDF-1b vector using NcoI and HindIII | This study |

| | | | |
|---|---|---|---|
| pSS27 | pCDF-1b | PCR amplification of *aca6* gene from gBlock and restriction cloning of *aca6* gene into pCDF-1b vector using NcoI and HindIII | This study |
| pSS28 | pCDF-1b | PCR amplification of *aca7* gene from gBlock and restriction cloning of *aca7* gene into pCDF-1b vector using NcoI and HindIII | This study |
| pSS29 | pCDF-1b | PCR amplification of *aca9* gene from gBlock and restriction cloning of *aca9* gene into pCDF-1b vector using NcoI and HindIII | This study |
| pSS30 | pCDF-1b | PCR amplification of *aca10* gene from gBlock and restriction cloning of *aca10* gene into pCDF-1b vector using NcoI and HindIII | This study |
| pSS31 | pCDF-1b | PCR amplification of *acrIIA1* gene from gBlock and restriction cloning of *acrIIA!* gene into pCDF-1b vector using NcoI and HindIII | This study |
| pSS32 | pGR2 | Restriction cloning of wt-IR *acrIE1-acrIF3-acrIF5-aca1* promoter into pGR2 vector using SpeI and PstI | This study |
| pPF1530 | pPF1439 (*eyfp* reporter plasmid) | Template for amplification of wild type *acrIF8-aca2* promoter | (10) |
| pSS33 | pGR2 | PCR amplification of *acrIF8-aca2* promoter from pPF1530 and restriction cloning of promoter into pGR2 vector using SpeI and PstI | This study (10) |
| pSS34 | pGR2 | Restriction cloning of wt-IR *acrIIC3-acrIIC2-aca3* promoter into pGR2 vector using SpeI and PstI | This study |
| pSS35 | pGR2 | Restriction cloning of wt-IR *acrIF12-aca4* promoter into pGR2 vector using SpeI and PstI | This study |
| pSS36 | pGR2 | Restriction cloning of wt-IR *acrIF20-acrIF19-acrIF17-acrIF16-aca5* promoter into pGR2 vector using SpeI and PstI | This study |
| pSS37 | pGR2 | Restriction cloning of wt-IR *candidate-acrIC6-aca6-putative* promoter into pGR2 vector using SpeI and PstI | This study |
| pSS38 | pGR2 | Restriction cloning of wt-IR *acrIF11-aca7-putative* promoter into pGR2 vector using SpeI and PstI | This study |

| | | | |
|---|---|---|---|
| pSS39 | pGR2 | Restriction cloning of wt-IR *acrIF22-aca9* promoter into pGR2 vector using SpeI and PstI | This study |
| pSS40 | pGR2 | Restriction cloning of wt-IR *acrIC7-acrIC6-aca10* promoter into pGR2 vector using SpeI and PstI | This study |
| pSS41 | pGR2 | Restriction cloning of wt-IR *acrIIA4-acrIIA1* promoter into pGR2 vector using SpeI and PstI | This study |
| pSS42 | pGR2 | Restriction cloning of sc-IR *acrIE1-acrIF3-acrIF5-aca1* promoter into pGR2 vector using SpeI and PstI | This study |
| pPF1580 | pPF1439 (*eyfp* reporter plasmid) | Template for PCR amplification of sc-IR1 *acrIF8-aca2* promoter | (10) |
| pSS43 | pGR2 | PCR amplification of sc-IR1 *acrIF8-aca2* promoter from pPF1580 and restriction cloning of promoter into pGR2 vector using SpeI and PstI | This study (10) |
| pSS44 | pGR2 | Restriction cloning of sc-IR *acrIIC3-acrIIC2-aca3* promoter into pGR2 vector using SpeI and PstI | This study |
| pSS45 | pGR2 | Restriction cloning of sc-IR *acrIF12-aca4* promoter into pGR2 vector using SpeI and PstI | This study |
| pSS46 | pGR2 | Restriction cloning of sc-IR *acrIF20-acrIF19-acrIF17-acrIF16-aca5* promoter into pGR2 vector using SpeI and PstI | This study |
| pSS47 | pGR2 | Restriction cloning of sc-IR *candidate-acrIC6-aca6-putative* promoter into pGR2 vector using SpeI and PstI | This study |
| pSS48 | pGR2 | Restriction cloning of sc-IR *acrIF11-aca7-putative* promoter into pGR2 vector using SpeI and PstI | This study |
| pSS49 | pGR2 | Restriction cloning of sc-IR *acrIF22-aca9* promoter into pGR2 vector using SpeI and PstI | This study |
| pSS50 | pGR2 | Restriction cloning of sc-IR *acrIC7-acrIC6-aca10* promoter into pGR2 vector using SpeI and PstI | This study |

| | | | |
|---|---|---|---|
| pSS51 | pGR2 | Restriction cloning of sc-IR *acrIIA4-acrIIA1* promoter into pGR2 vector using SpeI and PstI | This study |

**Supplementary Table S7: List of Software**

| **Program** | **Source** | **Reference** |
|---|---|---|
| Graphpad Prism 8.0 | https://www.graphpad.com/scientific-software/prism/ | GraphPad Software, La Jolla, California USA |
| CD-HIT-EST | https://www.weizhongli-lab.org/cd-hit/ | (24) |
| PROKKA | https://139.80.3.3:8080/<br>DeepVirome Galaxy Server, University of Otago | (25) |
| Psi-BLAST | https://www.blast.ncbi.nlm.nih.gov/Blast.cgi | (26) |
| HMMER3.0 | https://www.ebi.ac.uk/Tools/hmmer/ | (27) |
| RStudio | https://www.rstudio.com/ | (28) |
| Meme-Suite 5.2.0 | https://www.meme-suite.org/meme_5.2.0/ | (29) |
| MUSCLE (v 3.19) and Fasttree from Geneious Prime (v2019.1.3) | https://www.geneious.com | (30,31) |
| PhyloT (v2) | https://phylot.biobyte.de/ | |
| Itol(v5) | https://www.itol.embl.de/ | (32) |

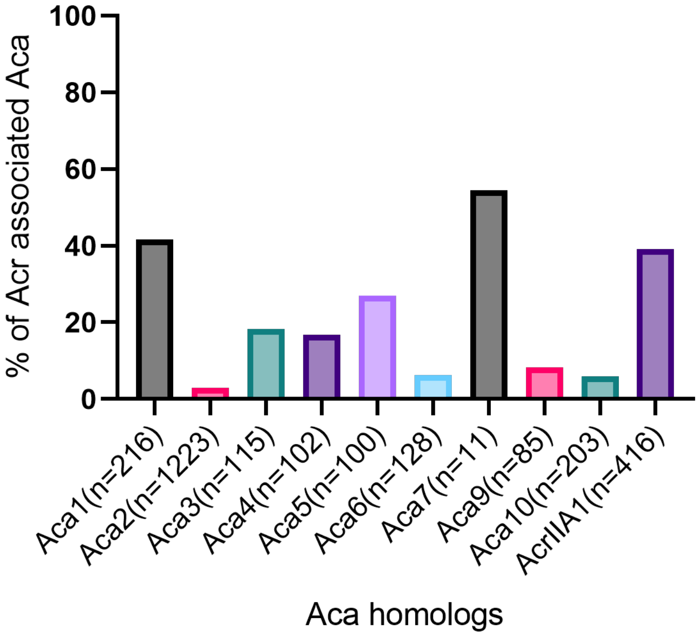


**Supplementary Figure S1**. Frequency of Acr-associated Aca homologs. The X-axis is the number of different Aca hits found by the HMM search and Y axis the percentage of these associated with an Acr (e.g. 40% of the 216 Aca1 homologs are Acr associated).

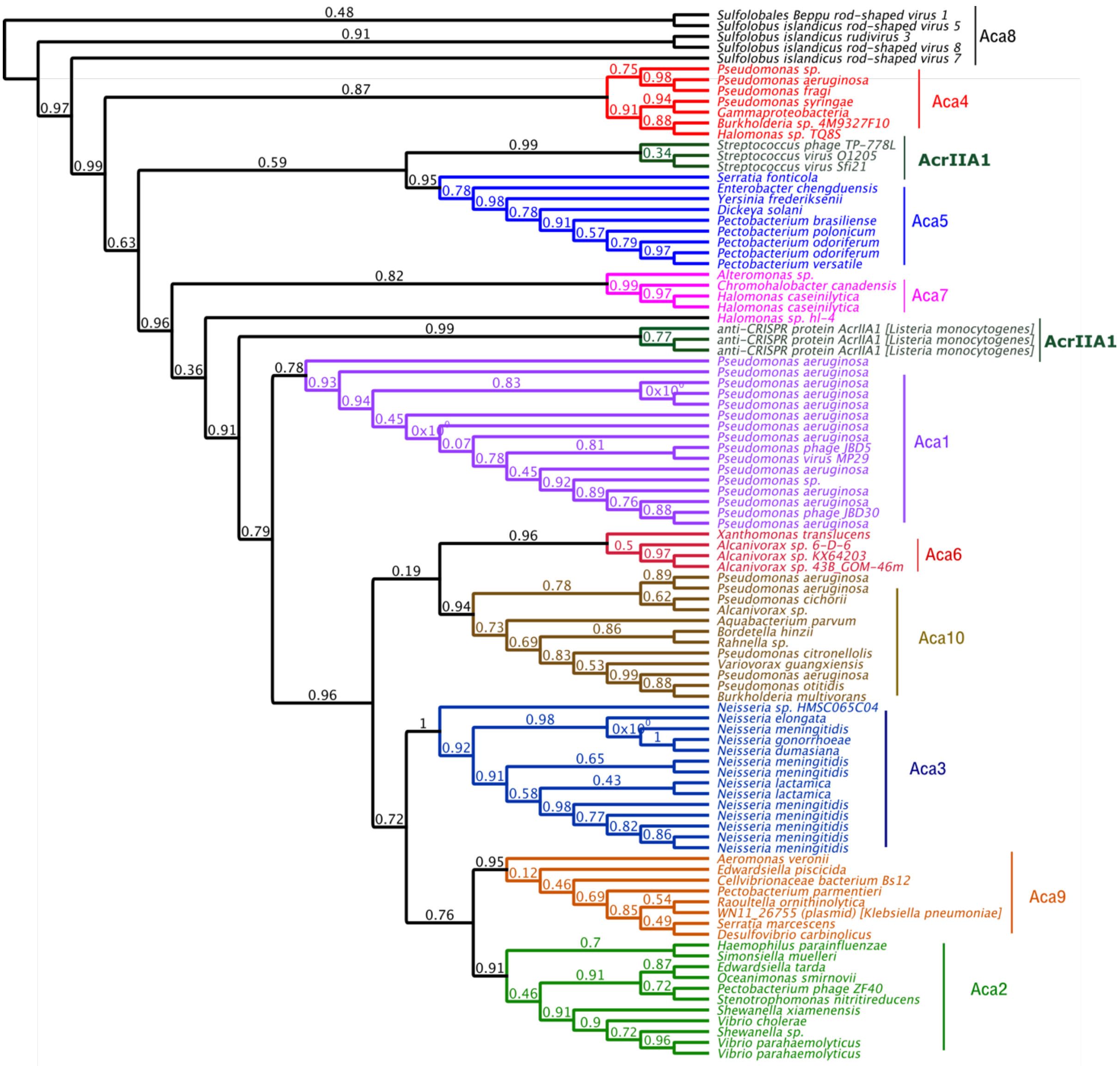


**Supplementary Figure S2.** Acas form distinct clades. Protein sequences were aligned with the MUSCLE algorithm by using the Neighbor-joining clustering method (maximum number of iterations: 500). The aligned sequences were then used to construct an approximately-maximum-likelihood tree using FastTree. Clades containing different Aca and AcrIIA1 are represented by different colours. The bootstrap values are shown beside nodes.

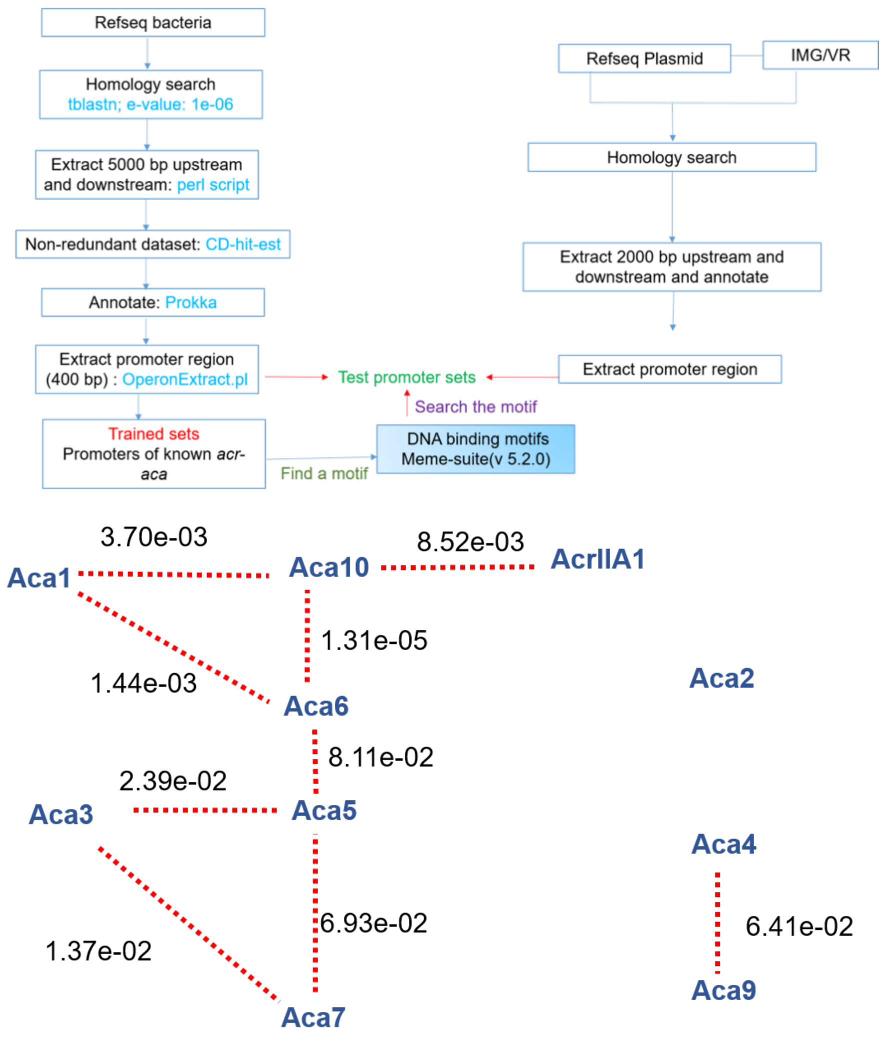


**Supplementary Figure S3.** Workflow for promoter analysis. The steps described in the Materials & Methods section are summarized. Comparison of different predicted Aca/AcrIIA1 binding motifs. The motifs were compared using TOMTOM (MEME-suite 5.2.0). Distances were measured using Pearson correlation coefficient. The red lines (with p-values) show the relation between the binding motifs that are predicted in the promoter regions for the respective Aca/AcrIIA1.

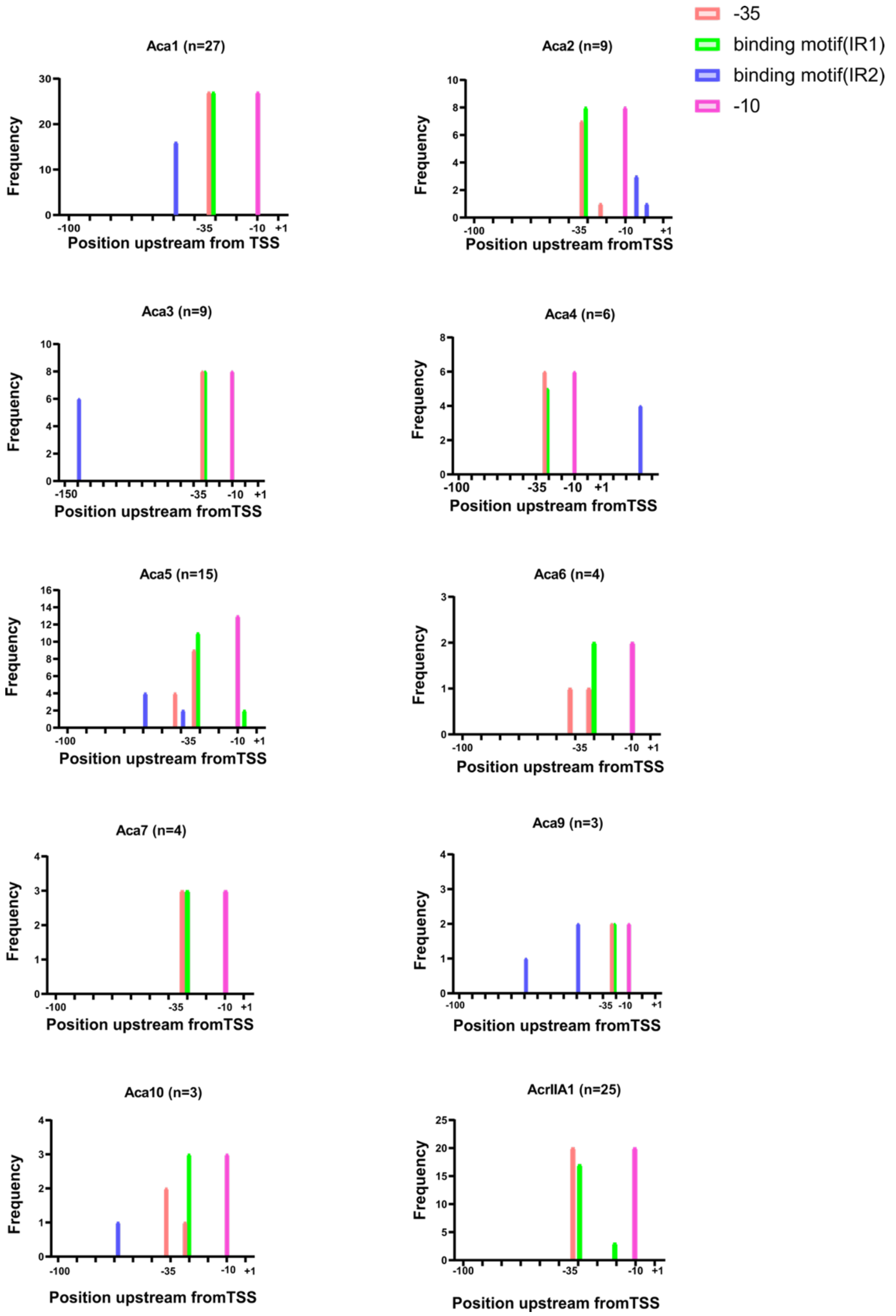


**Supplementary Figure S4.** Distribution of promoter elements and motifs in the high confidence datasets. Conserved elements such as -35 and -10 sequences were predicted in the putative *acr-aca* promoters. The predicted binding motifs (IR1 and IR2) are plotted relative to the -10 sequences. The transcription start sites (+1) of the downstream genes were calculated based on the position of -10 sequences. The X-axis and Y-axis respectively indicate the frequency and relative position of the binding sites.

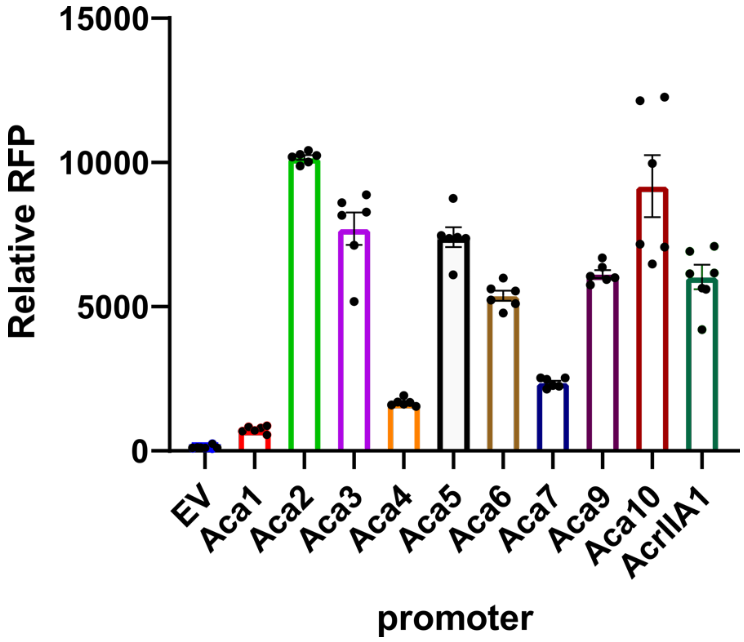


**Supplementary Figure S5**. Relative RFP fluorescence varies with different wild-type *acr-aca* promoters. Activity of the wild-type promoters in *E. coli* BL21 in the absence of Aca proteins, determined as relative RFP fluorescence. In each graph, the RFP fluorescence values were normalized with the $OD_{600}$ values. Data are presented as the mean ±SEM of six biological replicates.

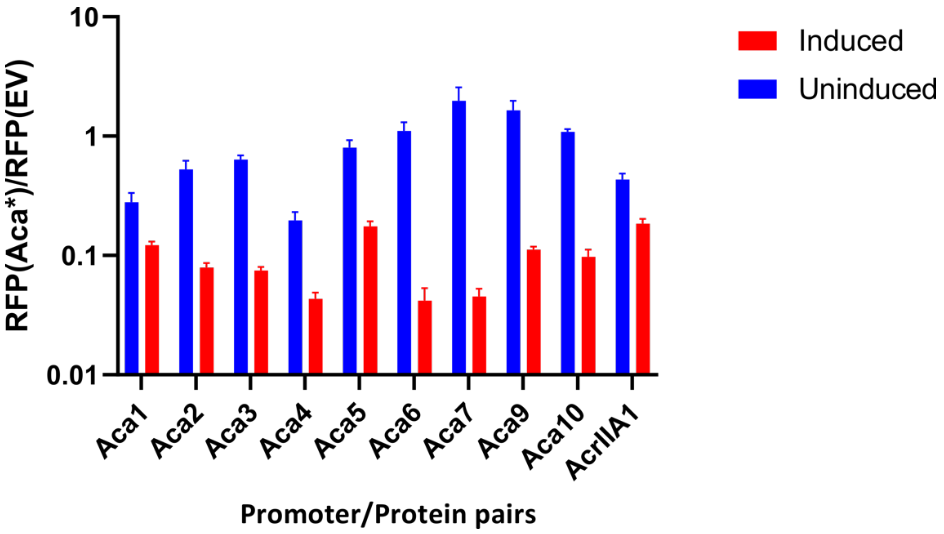


**Supplementary Figure S6**. Promoter repression is Aca-specific. The effect of the Aca proteins on cognate wild-type promoters in *E. coli* BL21 in the presence and absence of Aca proteins (± 50 µM IPTG), determined as relative RFP fluorescence. In each graph, the RFP fluorescence values were normalized with the $OD_{600}$ values. The relative RFP fluorescence with Aca proteins were divided by the RFP fluorescence without Aca. Thus, a value ≥1 means no repression or enhanced expression in the presence of the Aca protein, in comparison to values 0 or <1. Data are presented as the mean ±SEM of six biological replicates. * represents the corresponding Aca protein with its cognate promoter.

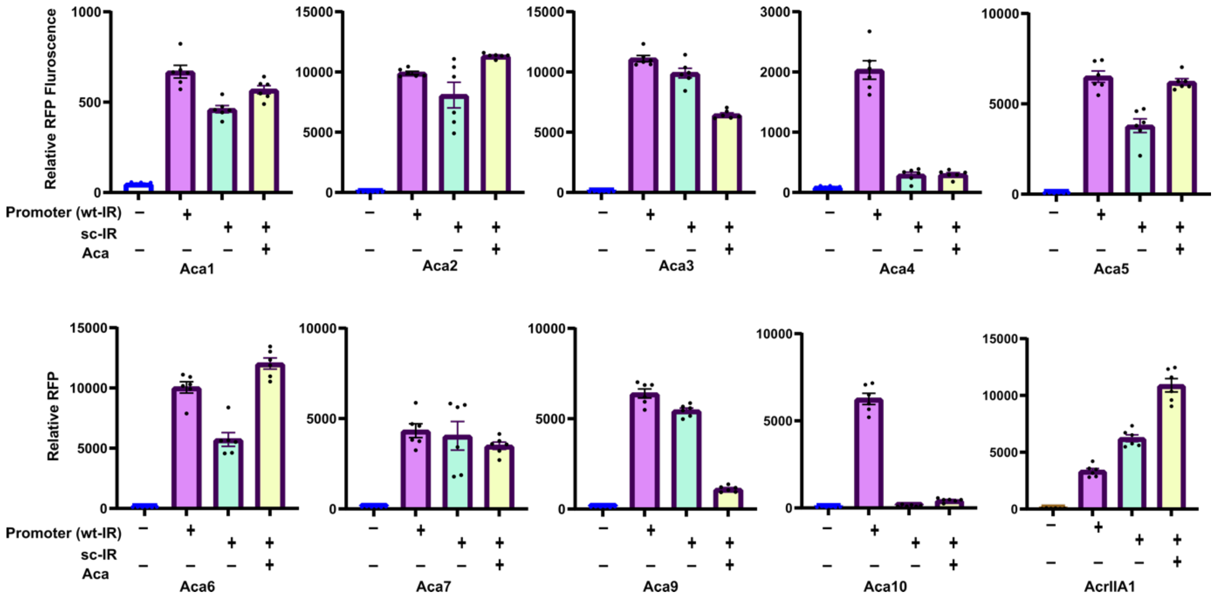


**Supplementary Figure S7**. Scrambling of the inverted repeats abolishes the effect of Aca proteins. Activity of the mutated promoter variants in *E. coli* BL21 in the presence and absence (+/-) of Aca proteins, determined as relative RFP fluorescence. In each graph, the RFP fluorescence values were normalized with the $OD_{600}$ values. Data are presented are the mean ±SEM of six biological replicates. In each case, + and - indicate presence or absence, respectively, of the promoters (wild type or scrambled) and *aca* genes.

## References


1. Bondy-Denomy, J., Pawluk, A., Maxwell, K.L. and Davidson, A.R. (2013) Bacteriophage genes that inactivate the CRISPR/Cas bacterial immune system. *Nature*, **493**, 429-432.
2. Pawluk, A., Bondy-Denomy, J., Cheung, V.H., Maxwell, K.L. and Davidson, A.R. (2014) A new group of phage anti-CRISPR genes inhibits the type IE CRISPR-Cas system of *Pseudomonas aeruginosa*. *MBio*, **5**.
3. Mahendra, C., Christie, K.A., Osuna, B.A., Pinilla-Redondo, R., Kleinstiver, B.P. and Bondy-Denomy, J. (2020) Broad-spectrum anti-CRISPR proteins facilitate horizontal gene transfer. *Nature microbiology*, **5**, 620-629.
4. Yin, Y., Yang, B. and Entwistle, S. (2019) Bioinformatics Identification of Anti-CRISPR Loci by Using Homology, Guilt-by-Association, and CRISPR Self-Targeting Spacer Approaches. *mSystems*, **4**, e00455-00419.
5. Gussow, A.B., Park, A.E., Borges, A.L., Shmakov, S.A., Makarova, K.S., Wolf, Y.I., Bondy-Denomy, J. and Koonin, E.V. (2020) Machine-learning approach expands the repertoire of anti-CRISPR protein families. *Nat Commun*, **11**, 3784.
6. Stanley, S.Y., Borges, A.L., Chen, K.H., Swaney, D.L., Krogan, N.J., Bondy-Denomy, J. and Davidson, A.R. (2019) Anti-CRISPR-Associated Proteins Are Crucial Repressors of Anti-CRISPR Transcription. *Cell*, **178**, 1452-1464 e1413.
7. Pawluk, A., Staals, R.H., Taylor, C., Watson, B.N., Saha, S., Fineran, P.C., Maxwell, K.L. and Davidson, A.R. (2016) Inactivation of CRISPR-Cas systems by anti-CRISPR proteins in diverse bacterial species. *Nat Microbiol*, **1**, 16085.
8. Liu, Y., Zhang, L., Guo, M., Chen, L., Wu, B. and Huang, H. (2021) Structural basis for anti-CRISPR repression mediated by bacterial operon proteins Aca1 and Aca2. *J. Biol. Chem.*, **297**, 101357.
9. Usher, B., Birkholz, N., Beck, I.N., Fagerlund, R.D., Jackson, S.A., Fineran, P.C. and Blower, T.R. (2021) Crystal structure of the anti-CRISPR repressor Aca2. *J. Struct. Biol.*, **213**, 107752.
10. Birkholz, N., Fagerlund, R.D., Smith, L.M., Jackson, S.A. and Fineran, P.C. (2019) The autoregulator Aca2 mediates anti-CRISPR repression. *Nucleic Acids Res.*, **47**, 9658-9665.

11. Brown, B.L., Lord, D.M., Grigoriu, S., Peti, W. and Page, R. (2013) The *Escherichia coli* toxin MqsR destabilizes the transcriptional repression complex formed between the antitoxin MqsA and the mqsRA operon promoter. *J. Biol. Chem.*, **288**, 1286-1294.
12. Marino, N.D., Zhang, J.Y., Borges, A.L., Sousa, A.A., Leon, L.M., Rauch, B.J., Walton, R.T., Berry, J.D., Joung, J.K., Kleinstiver, B.P. *et al.* (2018) Discovery of widespread type I and type V CRISPR-Cas inhibitors. *Science*, **362**, 240-242.
13. Kostelidou, K., Jones, A.C. and Thomas, C.M. (1999) Conserved C-terminal region of global repressor KorA of broad-host-range plasmid RK2 is required for co-operativity between KorA and a second RK2 global regulator, KorB. *J. Mol. Biol.*, **289**, 211-221.
14. Klimuk, E., Bogdanova, E., Nagornykh, M., Rodic, A., Djordjevic, M., Medvedeva, S., Pavlova, O. and Severinov, K. (2018) Controller protein of restriction-modification system Kpn2I affects transcription of its gene by acting as a transcription elongation roadblock. *Nucleic Acids Res.*, **46**, 10810-10826.
15. Pinilla-Redondo, R., Shehreen, S., Marino, N.D., Fagerlund, R.D., Brown, C.M., Sorensen, S.J., Fineran, P.C. and Bondy-Denomy, J. (2020) Discovery of multiple anti-CRISPRs highlights anti-defense gene clustering in mobile genetic elements. *Nat Commun*, **11**, 5652.
16. Hadzi, S., Garcia-Pino, A., Haesaerts, S., Jurenas, D., Gerdes, K., Lah, J. and Loris, R. (2017) Ribosome-dependent *Vibrio cholerae* mRNAse HigB2 is regulated by a beta-strand sliding mechanism. *Nucleic Acids Res.*, **45**, 4972-4983.
17. Leon, L.M., Park, A.E., Borges, A.L., Zhang, J.Y. and Bondy-Denomy, J. (2021) Mobile element warfare via CRISPR and anti-CRISPR in *Pseudomonas aeruginosa*. *Nucleic Acids Res.*, **49**, 2114-2125.
18. Pawluk, A., Amrani, N., Zhang, Y., Garcia, B., Hidalgo-Reyes, Y., Lee, J., Edraki, A., Shah, M., Sontheimer, E.J., Maxwell, K.L. *et al.* (2016) Naturally Occurring Off-Switches for CRISPR-Cas9. *Cell*, **167**, 1829-1838 e1829.
19. Osuna, B.A., Karambelkar, S., Mahendra, C., Christie, K.A., Garcia, B., Davidson, A.R., Kleinstiver, B.P., Kilcher, S. and Bondy-Denomy, J. (2020) *Listeria* Phages Induce Cas9 Degradation to Protect Lysogenic Genomes. *Cell Host Microbe*, **28**, 31-40 e39.
20. Osuna, B.A., Karambelkar, S., Mahendra, C., Sarbach, A., Johnson, M.C., Kilcher, S. and Bondy-Denomy, J. (2020) Critical Anti-CRISPR Locus Repression by a Bi-functional Cas9 Inhibitor. *Cell Host Microbe*, **28**, 23-30 e25.
21. Talavera, A., Tamman, H., Ainelo, A., Konijnenberg, A., Hadzi, S., Sobott, F., Garcia-Pino, A., Horak, R. and Loris, R. (2019) A dual role in regulation and toxicity for the disordered N-terminus of the toxin GraT. *Nat Commun*, **10**, 972.
22. Taylor, R.G., Walker, D.C. and McInnes, R.R. (1993) *E. coli* host strains significantly affect the quality of small scale plasmid DNA preparations used for sequencing. *Nucleic Acids Res.*, **21**, 1677-1678.
23. Studier, F.W. and Moffatt, B.A. (1986) Use of bacteriophage T7 RNA polymerase to direct selective high-level expression of cloned genes. *J. Mol. Biol.*, **189**, 113-130.
24. Huang, Y., Niu, B., Gao, Y., Fu, L. and Li, W. (2010) CD-HIT Suite: a web server for clustering and comparing biological sequences. *Bioinformatics*, **26**, 680-682.
25. Seemann, T. (2014) Prokka: rapid prokaryotic genome annotation. *Bioinformatics*, **30**, 2068-2069.
26. Altschul, S.F., Madden, T.L., Schaffer, A.A., Zhang, J., Zhang, Z., Miller, W. and Lipman, D.J. (1997) Gapped BLAST and PSI-BLAST: a new generation of protein database search programs. *Nucleic Acids Res.*, **25**, 3389-3402.
27. Potter, S.C., Luciani, A., Eddy, S.R., Park, Y., Lopez, R. and Finn, R.D. (2018) HMMER web server: 2018 update. *Nucleic Acids Res.*, **46**, W200-W204.
28. Team, R. (2021). RStudio: integrated development for R. RStudio, PBC, Boston, MA. 2020
29. Bailey, T.L., Johnson, J., Grant, C.E. and Noble, W.S. (2015) The MEME Suite. *Nucleic Acids Res.*, **43**, W39-49.
30. Price, M.N., Dehal, P.S. and Arkin, A.P. (2010) FastTree 2–approximately maximum-likelihood trees for large alignments. *PLoS One*, **5**, e9490.
31. Edgar, R.C. (2004) MUSCLE: a multiple sequence alignment method with reduced time and space complexity. *BMC Bioinformatics*, **5**, 113.

32. Letunic, I. and Bork, P. (2021) Interactive Tree Of Life (iTOL) v5: an online tool for phylogenetic tree display and annotation. *Nucleic Acids Res.*, **49**, W293-W296.

## Figures and Tables captions

Supplementary Table S1: Chosen parameters for psi-blast

Supplementary Table S2: Number of Aca-like proteins analysed, those with >40% query coverage from Figure S1)

Supplementary Table S3: Number of promoter sequences for the analyses

Supplementary Table S4: List of oligonucleotides used in this study

Supplementary Table S5: Bacterial strains used in this study

Supplementary Table S6: Plasmids used in this study

Supplementary Table S7: List of Software

**Supplementary Figure S1**. Frequency of Acr-associated Aca homologs. The X-axis is the number of different Aca hits found by the HMM search and Y axis the percentage of these associated with an Acr (e.g. 40% of the 216 Aca1 homologs are Acr associated).

**Supplementary Figure S2.** Acas form distinct clades. Protein sequences were aligned with the MUSCLE algorithm by using the Neighbor-joining clustering method (maximum number of iterations: 500). The aligned sequences were then used to construct an approximately-maximum-likelihood tree using FastTree. Clades containing different Aca and AcrIIA1 are represented by different colours. The bootstrap values are shown beside nodes.

**Supplementary Figure S3.** Workflow for promoter analysis. The steps described in the Materials & Methods section are summarized. Comparison of different predicted Aca/AcrIIA1 binding motifs. The motifs were compared using TOMTOM (MEME-suite 5.2.0). Distances were measured using Pearson correlation coefficient. The red lines (with p-values) show the relation between the binding motifs that are predicted in the promoter regions for the respective Aca/AcrIIA1.

**Supplementary Figure S4.** Distribution of promoter elements and motifs in the high confidence datasets. Conserved elements such as -35 and -10 sequences were predicted in the putative *acr-aca* promoters. The predicted binding motifs (IR1 and IR2) are plotted relative to the -10 sequences. The transcription start sites (+1) of the downstream genes were calculated based on the position of -10 sequences. The X-axis and Y-axis respectively indicate the frequency and relative position of the binding sites.

**Supplementary Figure S5.** Relative RFP fluorescence varies with different wild-type *acr-aca* promoters. Activity of the wild-type promoters in *E. coli* BL21 in the absence of Aca proteins, determined as relative RFP fluorescence. In each graph, the RFP fluorescence values were normalized with the $OD_{600}$ values. Data are presented as the mean ±SEM of six biological replicates.

**Supplementary Figure S6.** Promoter repression is Aca-specific. The effect of the Aca proteins on cognate wild-type promoters in *E. coli* BL21 in the presence and absence of Aca proteins (± 50 µM IPTG), determined as relative RFP fluorescence. In each graph, the RFP fluorescence values were normalized with the $OD_{600}$ values. The relative RFP fluorescence with Aca proteins were divided by the RFP fluorescence without Aca. Thus, a value ≥1 means no repression or enhanced expression in the presence of the Aca protein, in comparison to values 0 or <1. Data are presented as the mean ±SEM of six biological replicates. * represents the corresponding Aca protein with its cognate promoter.

**Supplementary Figure S7.** Scrambling of the inverted repeats abolishes the effect of Aca proteins. Activity of the mutated promoter variants in *E. coli* BL21 in the presence and absence (+/-) of Aca proteins, determined as relative RFP fluorescence. In each graph, the RFP fluorescence values were normalized with the $OD_{600}$ values. Data are presented are the mean ±SEM of six biological replicates. In each case, + and - indicate presence or absence, respectively, of the promoters (wild type or scrambled) and *aca* genes.
